# Connectedness With Nature and Individual Responses to a Pandemic: An Exploratory Study

**DOI:** 10.3389/fpsyg.2020.02215

**Published:** 2020-09-23

**Authors:** Simona Haasova, Sandor Czellar, Leïla Rahmani, Natalie Morgan

**Affiliations:** Department of Marketing, University of Lausanne, Lausanne, Switzerland

**Keywords:** pandemic, health, nature, connectedness with nature, attitudes, behaviors

## Abstract

Most recent epidemics have originated in complex human-nature interactions and yet, our knowledge is very limited regarding the psychological aspects of human-nature relationships that underlie individual human responses in times of pandemic crises. We propose that the concept of connectedness with nature and associated individual difference measures offer a relevant and useful lens to inform us about how humans think, feel and behave in such critical times. Our two-wave study, conducted with 486 United States residents at the end of March 2020 (wave 1) and 533 United States residents at the beginning of May 2020 (wave 2), focuses on the 2020 coronavirus situation. It maps individual responses to the current pandemic in terms of mental representations, behavioral tendencies and perceived impact, and explores the relationships of these constructs to individual levels of connectedness with nature. As this research employs an exploratory methodology, our results provide an account of potential relationships rather than their validation and thus represent an encouraging steppingstone for research on human behavior in the time of a global pandemic. We identify a series of research propositions and questions for systematic future inquiry.

## Introduction

As of March 2020, coronavirus is the new reality for almost every human being living on planet Earth (Garfin et al., 2020; Perlman, 2020; Sohrabi et al., 2020). The dangers and the unknown that the COVID-19 pandemic carries for the global population warrant scientific efforts to focus on the accumulation of knowledge that can inform the implementation of effective measures to confront it; all the more so that if viral pandemics of such a scale may also erupt in the future (Di Marco et al., 2020). As social scientists, we would like to contribute to these efforts and fast track our understanding of the factors pertinent to human perceptions, attitudes and actions during pandemics.

The present research is a step in this direction, having two particular aims in mind. Viewing the current pandemic on a larger scale, it most likely has its origins in nature (specifically, wild animals) and the societal attempt to contain it constitutes, at its core, an issue of global human-nature interaction (Zhou et al., 2020). Therefore, the first aim of the present research is to utilize the lens of the human-nature relationship to investigate individual psychological responses to a pandemic. To that effect, we focus on mapping how the connection between people and their natural environment relates to their: (1) individual representations about the coronavirus pandemic; (2) relevant behaviors during the pandemic; and (3) perceptions of impact of the pandemic on their own lives and other people. The present study contributes to the current body of research on human behavior in pandemic-like situations by providing, to our knowledge, the first comprehensive account of the psychological dimensions of human reactions to a global pandemic and how these reactions are intertwined with the connection people have to the natural environment.

The COVID-19 pandemic represents a naturally occurring yet infrequent event. The potential insights that the concept of connectedness with nature may provide about relevant psychological responses constitute an open research question that has not been previously investigated. Therefore, we address it in the present research with an exploratory study, a methodological approach that is warranted for the investigation of novel research questions, i.e., questions for which there is not sufficient scientific evidence as of yet to warrant the development and test of clearly articulated hypotheses (Creswell, 2009; Sarantakos, 2013). This broader approach allows us to develop a better initial understanding of the research domain at hand and provides a rich platform for future investigations (Hartmann and Hedblom, 1979; Stebbins, 2001; Creswell, 2009; Sarantakos, 2013). Relatedly, the second aim of the current research is to use our exploratory data for the construction of a series of research propositions and questions, based on our empirical exploration of the potential relationships between individuals’ connection to nature and their responses to a pandemic. The research propositions and questions thus generated constitute a roadmap for further environmental research and foster theory development on the important and timely topic of the role of human-nature relationships in human reactions to a pandemic.

### The Concept of Connectedness With Nature and Its Significance in Pandemic Times

The concept of connectedness with nature, also referred to as self-nature connection or nature connectedness, is defined as “the extent to which an individual includes nature within his/her cognitive representation of self” (Schultz, 2002, p. 67). It is embedded within the broader research paradigm of individual environmental identity, which investigates how humans, in their daily lives, rely on identity-based motivations to manage their attitudes, relations, and behaviors toward the natural environment (Clayton, 2003, 2012). Researchers have developed several valuable scales for the assessment of relevant aspects of the connectedness with nature construct (e.g., Schultz, 2001; Mayer and Frantz, 2004; Martin and Czellar, 2016; Richardson et al., 2019). Its psychological and behavioral correlates have also received considerable scholarly attention in recent years. First-hand direct experiences with the natural environment seem influential in shaping individual connectedness with nature (e.g., Cheng and Monroe, 2012; Collado et al., 2015). The latter has been shown to meaningfully relate to the tendency to espouse biospheric motivations and values as well as pro-environmental attitudes (e.g., Mayer and Frantz, 2004; Martin and Czellar, 2017). If a person includes the natural environment as part of their self-view, they are more likely to engage in nature-protective behaviors as well (Schultz, 2002; Mayer and Frantz, 2004). Indeed, connectedness with nature is positively related to self-reported and actual pro-environmental behavior, as summarized in recent meta-analyses on the topic (Mackay and Schmitt, 2019; Whitburn et al., 2020). Additionally, empirical evidence shows that connectedness with nature is positively associated with subjective perceptions of happiness, well-being, and general satisfaction with life (e.g., Mayer and Frantz, 2004; Howell et al., 2011; Zelenski and Nisbet, 2014).

Thus, extant research suggests that the concept of self-nature connection captures the multi-faceted links humans nurture with their natural surroundings. The way humans relate to the natural environment arguably presents a highly relevant lens for the study of human behavior during events originating in human-nature interactions (Clayton and Opotow, 2003). The purpose of our current investigation is to learn more about how connectedness with nature relates to psychological responses to a global epidemic that likely originates in complex human-nature interactions as well (Zhou et al., 2020). We are particularly interested to find out more about the individual representations, behavioral reactions and perceptions of impact that individuals entertain about the pandemic, and how these different psychological tendencies are associated with higher (vs. lower) levels of self-nature connection. Nevertheless, because of the unprecedented scale and amplitude of the current pandemic and lack of a relevant integrative conceptual framework, how self-nature connection shapes individual perceptions and reactions to, and is in turn affected by, such a global crisis remains a largely unexplored research domain. In the current state of our knowledge and as illustrated below, alternative, and often opposing predictions could be formulated about how nature connectedness links to individual representations about, behaviors toward, and perceptions relating to the potential impact of the pandemic.

#### Individual Representations About the Pandemic and Connectedness With Nature

On the one hand, a close bond with nature may lead people to view the pandemic as a result of yet another series of unsustainable human actions. Anthropogenic activity, such as deforestation, and a rise in human population has indeed been linked to the increase in emerging infectious diseases (Jones et al., 2008; Di Marco et al., 2020). The coronavirus pandemic itself has been suggested to be related to humanity’s excessive intrusion into nature and illegal wildlife trade (Weston, 2020). Consequently, this may lead to pronounced negative views about the pandemic among people who strongly connect with nature. On the other hand, given that the sources of the pandemic are likely to ultimately originate in nature (Zhou et al., 2020), a stronger connection to nature may relate to a higher likelihood of ascribing natural sources to the pandemic. As a result, individuals with a stronger (vs. weaker) connectedness with nature may have less negative representations about the coronavirus.

Meanwhile, the media increasingly report alleged improvements of environmental conditions associated with climate change as a direct result of the lockdown regulations, such as a decrease in air pollution or carbon footprint (Okyere et al., 2020). These improvements usually represent an important goal for pro-environmental individuals, which might contribute to their awareness of these issues and attitudes regarding the positive aspects of the pandemic. However, while we urgently need to reduce our CO_2_ emissions, a pandemic is not a solution to climate change. The emission reductions in the second quarter of 2020 are the result of temporary measures taken to curb the spread of the coronavirus and we do not know whether long-term conservational regulations will ensue after this health crisis (Link, 2020). Another concern is that the fight against the virus has become the priority of most countries, and although understandable, this has led to discussions on climate change being relegated to the background (Ambrose and Harvey, 2020). These latter tendencies may, in turn, negatively impact attitudes about the pandemic for individuals with strong (vs. weak) self-nature connection.

#### Individual Behavior During the Pandemic and Connectedness With Nature

With medical remedies still mostly under development (Cascella et al., 2020; Sohrabi et al., 2020), much relies on individuals taking action to prevent further transmission of the virus. The primary objectives of the international community relate to the circumvention of disease transmission at a global level through a series of public health and safety recommendations (World Health Organization, 2020). The protective measures include frequent hand washing, wearing a mask, staying informed and social distancing. Possibly, people with a more pronounced connectedness with nature may comply more with these measures, and perceive their actions as more effective, because they may want to quickly bring an end to the crisis in order to remain protective of nature, which, broadly defined, may include all living beings and therefore humans as well (Clayton, 2003). On the other hand, they could also be more reluctant to do so because they might see an epidemic originating in nature as part of the “web of life” (Mayer and Frantz, 2004) that should not warrant excessive human intervention. It could also be that people with a higher (vs. lower) nature connectedness may see the official measures to be too restrictive of their opportunities of regular contact with nature (Collado et al., 2015).

Regarding personal movement, individuals around the globe are asked to practice self-quarantine, limiting activities outside of their homes only to the bare necessities, minimizing the use of public spaces, traveling and transportation in general. People with a stronger (vs. weaker) connection with nature may more easily comply with these measures as they could be more likely to see the pro-environmental benefits of limited movement. However, the opposite may also hold: people with a stronger (vs. weaker) nature connectedness may comply less with the movement restrictions because going outside to forests, rivers and lakes, and in general, engaging in nature-related activities may help them benefit from the restorative power of natural spaces (Hartig et al., 2014; Collado et al., 2017).

#### Individual Perceptions About the Impact of the Pandemic and Connectedness With Nature

To what extent do individuals believe that they are more, or less, likely to be closely threatened and affected by the virus? This question is highly relevant in the current context because the perception of an event as being psychologically close rather than distant, whether socially, temporarily, geographically, or hypothetically, is associated with higher preparedness to act and engage in relevant behaviors (Trope and Liberman, 2010). Individuals may be less likely to comply with protective measures if they believe that they are less likely to be threatened and affected personally by the virus. How pronouncedly individuals experience themselves to be a part of the broader natural world, potentially inclusive of other human beings, may be associated with the perception of whether the coronavirus could directly impact oneself, together with or apart from others. On the one hand, we could speculate that more pronounced perceptions of connectedness with nature lead to stronger feelings of being part of the “web of life” (Mayer and Frantz, 2004), which in turn could suggest the inference that natural events—including a pandemic—are just as likely to reach oneself as other humans. On the other hand, the opposite prediction also seems reasonable. An increased sense of experiencing connections with nature has been shown to be associated with a more balanced diet, overall health and general well-being (Howell et al., 2011; Gill, 2014; Sobko et al., 2020). People with such tendencies may therefore perceive themselves to be relatively fitter and therefore less vulnerable to face events with aversive health impact such as a viral pandemic. Relatedly, it could also be that people who are more connected to nature feel that they are comparatively less exposed to the risks of the illness than others who are less connected with nature.

In sum, the relations between individual nature connectedness and responses to a pandemic situation (specifically individual representations, behavioral tendencies and perceptions of impact of the pandemic on people’s lives) may be complex and multi-faceted, constituting research questions that have so far received little theoretical and empirical attention in the literature. We therefore conducted a study of an *exploratory nature*, leaning on the discovery of potential relationships between multiple constructs pertaining to peoples’ psychological reactions to the pandemic and their connection to nature, rather than on the validation of *a priori* hypotheses built on extant research. This is due to the novelty and complexity of the situation in people’s life experiences; and to the lack of a comprehensive conceptual framework linking individual characteristics related to their natural environment with the specifics of a pandemic situation. We provide a detailed overview of the potential relationships between individual psychological responses to a pandemic and nature connectedness grouped around three themes: individual representations about the pandemic, behavioral tendencies, and perceived impact of the pandemic. Based on these exploratory insights, we then extract and provide formal research questions and testable propositions for future research about the relation between connectedness to nature and human reactions to pandemics.

## Materials and Methods

In this exploratory study, we examine whether the concept of connectedness with nature can help us better understand people’s perceptions, attitudes, and actions in a pandemic situation. We aim to map how individuals view a pandemic, engage in relevant individual behaviors and perceive the impact of the pandemic on their and other’s lives. Most importantly, we intend to lay out potential relationships between these factors and individual self-nature connection. In order to increase the consistency and stability of our findings over time, we administered a survey in two waves and collected the data at two time points during the coronavirus pandemic, five weeks apart. To expand its exploratory and convergence potential, we included in the survey several measures of concepts related to self-nature connection as well as a series of attitudinal, perceptual and behavioral measures linked to pro-environmentalism, self-concept and dimensions of psychological distance, all pertaining to the ongoing pandemic. Additionally, the study also included some measures whose focus was not on the pandemic and are thus not reported on further. The measures on which we report in the paper and in the Supplementary Material were identical for both waves of data collection. The exhaustive list of measures, their sources, concrete items and response formats, including scale reliability statistics and descriptive statistics, can be found in the Supplementary Material. Both waves of the study were separately pre-registered prior to data collection, explicitly stating its exploratory, and not hypothesis-testing, purpose—the pre-registration documents can be found under these links: https://aspredicted.org/69an2.pdf and https://aspredicted.org/ys3f3.pdf. The original contributions presented in the study are publicly available. This data can be found here: https://drive.switch.ch/index.php/s/9AcRbu4QicYjSn5.

From a methodological perspective, we intended to account for two presumed causes of common method variance, namely, common scale format and social desirability (Podsakoff et al., 2003). By employing different scale formats in our measurements, we followed Podsakoff et al. (2003) recommendations for procedural remedies to avoid a common scale format that could, due to the correlational and proximal nature of our measurements, possibly inflate the potential relationships between the assessed variables. In order to minimize order effects, we have also randomized the order of measures and items whenever possible. By measuring and statistically correcting for individual levels of social desirability in our analyses (e.g., partial correlations accounting for the effect of social desirability), we followed the recommendations of Siemsen et al. (2010) and Simmering et al. (2015).

### Participants

In the first (second) wave, a total of 563 (646) United States residents participated in an M-Turk survey in exchange for a payment of $1.20. This first-wave data was collected on Saturday, March 28th, 2020, at the accelerating stage of the pandemic in the United States—by that day, the United States had recorded a total of 118766 infections and 2754 deaths due to the coronavirus (Worldometer, 2020). The second wave of data collection took place on Saturday, May 2nd, 2020, when the toll had reached 931370 infections and 68597 recorded deaths (Worldometer, 2020). Federal coronavirus guidelines, including recommendations for social distancing, stay-at-home orders, hand-washing and staying informed were issued by the White House on March 16, 2020 (White House, 2020) and thus were in place at both times of our data collection.

Following our pre-registered criteria, we have excluded those participants who did not completely finish the questionnaire (wave_1_ = 56; wave_2_ = 81), those who indicated that they did not wish their data to be included in the data analyses (wave_1_ = 8; wave_2_ = 6) and then those who did not pass the attention check (wave_1_ = 13; wave_2_ = 26). The final sample of wave 1 consisted of 486 participants, from which 46.5% were women (1.6% did not wish to disclose this information), with a mean age of 39.59 years (*SD* = 13.11) and the three most frequent states of origin were California (52), Texas (33), and New York (30). The final sample of wave 2 consisted of 533 participants, from which 44.7% were women (0.8% did not wish to disclose this information), with a mean age of 39.44 years (*SD* = 13.35) and the three most frequent states of origin were California (51), Texas (49), and Florida (44). All other characteristics describing both samples in depth are presented in Table 1. Our samples seem fairly representative of the United States population as of 2018 (United States Census Bureau, 2020) in terms of gender (female_wave1_ = 46.5%; female_wave2_ = 46.5%; female_United States 2018_ = 50.8%), age (median_wave1_ = 37.0; median_wave2_ = 35.0; median_United States 2018_ = 38.2), and somewhat also in annual household income (median_wave1_ income category $50000–$59999; median_wave2_ income category $50000–$59999; median_United States 2018_ = $ 61.937). However, our sample tended to be more educated in comparison with the national statistic (92.2% of wave 1 and 93.8% of wave 2 reported education higher than high school vs. 61.5% in the United States population of 2018).

**TABLE 1:** Characteristics of the participants (wave 1: *N* = 486; wave 2: *N* = 533).

Characteristic	Percent (Wave 1)	Percent (Wave 2)
**Gender**		
Female	46.6	44.7
Male	51.8	55.0
Not reported	1.6	0.8
**Educational level**		
Less than High School	–	0.9
High School/GED	7.8	5.3
Some college	19.9	15.2
2nd year college	8.4	9.0
4th year college	43.1	48.4
Masters	17.7	17.1
Ph.D. degree	1	2.4
Prof. degree	2.1	1.7
**State**		
Alabama	0.8	0.4
Alaska	0.2	0.2
Arizona	2.3	2.3
Arkansas	1.0	0.6
California	10.7	9.6
Colorado	2.5	2.1
Connecticut	0.6	1.5
Delaware	–	0.6
Florida	5.7	8.3
Georgia	2.3	2.3
Hawaii	0.6	0.4
Idaho	0.4	0.4
Illinois	2.5	4.3
Indiana	1.8	0.9
Iowa	0.6	1.1
Kansas	1.2	1.1
Kentucky	0.6	1.1
Louisiana	1.2	0.8
Maine	0.4	0.2
Maryland	1.4	2.6
Massachusetts	2.5	1.3
Michigan	2.7	2.6
Minnesota	0.6	0.8
Mississippi	0.2	0.6
Missouri	1.6	1.5
Montana	0.4	0.2
Nebraska	0.2	0.2
Nevada	1.0	1.3
New Hampshire	0.2	0.6
New Jersey	2.3	1.3
New Mexico	0.6	0.6
New York	6.2	5.8
North Carolina	1.0	4.5
Ohio	2.3	1.9
Oklahoma	0.8	1.3
Oregon	1.2	0.8
Pennsylvania	3.1	2.4
Rhode Island	–	0.6
South Carolina	1.0	0.4
South Dakota	0.4	0.6
Tennessee	1.8	1.7
Texas	6.8	9.2
Utah	0.8	0.9
Virginia	2.1	2.1
Washington	3.5	2.6
West Virginia	1.0	0.4
Wisconsin	2.1	1.3
Wyoming	0.2	0.6
Not reported	0.2	–
**Living area**		
Urban	33.5	38.3
Rather urban	34.7	36.1
Rural	9.4	14.5
Rather rural	22.2	11.1
**Net annual income combined ($)**		
< 30000	16.8	16.1
30000–39999	12.5	10
40000–49000	9	13.8
50000–59999	11.7	12.8
60000–69999	10.1	9.0
70000–79999	11.5	8.4
80000–89999	6	7.7
90000–99999	6.2	5.7
> 100000	13.3	16.1
Not reported	2.9	2.5

### Design and Procedure

The study’s design and procedure were similar for both waves of data collection. After providing informed consent, the data was collected in two measurement blocks, with the order counterbalanced between participants.

In block one, we assessed self-nature connection with the Extended Inclusion of Nature in Self scale (Martin and Czellar, 2016) and Nature Connection Index (Richardson et al., 2019), followed by a measure of Green Self-Identity (Sparks and Shepherd, 1992). The measures in this block were presented in a random order.

In block two, we first asked participants about their general and self-related perceptions and beliefs of the coronavirus pandemic. In a free word association task (adapted from Lorenzoni et al., 2006), participants were asked to write down all the words that came into their mind about the term “coronavirus” and subsequently evaluate them on valence. We assessed perception of threat (adapted from Bord et al., 1998) and perception of the potentially good and bad things that have come with the pandemic (adapted from Spence et al., 2010). Two external judges (blind to the purposes of the study) categorized the associations and the positive/negative aspects participants reported about the pandemic into common themes according to their frequency; any disagreements were resolved upon discussion. Beliefs about the causes (nature vs. human activity) of the pandemic, as well as beliefs about personal self-efficacy in handling the pandemic situation and preparedness to act (all three measures adapted from Heath and Gifford, 2006) were also measured.

Then, we asked how the pandemic impacted personal lives and lives of others with a series of questions pertaining to the experience on four dimensions of psychological distance toward the pandemic (Trope and Liberman, 2010). We measured how much participants thought that the pandemic affected local and distant areas (geographical distance), and people similar or different to oneself (social distance; both adapted from Spence et al., 2012). Furthermore, participants reported when they thought their personal and others’ lives would be affected by the virus (temporal distance; adapted from Spence et al., 2012) and how they gauged the likelihood of it being contracted by themselves and others (hypothetical distance). We also asked about their familiarity with the closest person infected by the virus.

Next, we inquired about behavioral tendencies, i.e., participant’s self-reported behaviors and compliance with the health safety measures. We asked what nature-related and nature-unrelated activities people found themselves doing more during the pandemic times than usual. Participants then reported the extent to which they complied with the global health safety measures (e.g., social distancing), whether they practiced self-quarantine and for how many days. We then assessed participants’ beliefs in a universal, higher-order meaning underlying the cause of the pandemic.

Lastly, we assessed individual tendency to behave in a socially desirable manner (adapted from Hart et al., 2015), and collected demographic information. An attention check and data inclusion questions ended the survey.

In the following sections, we present descriptive statistics on a series of variables grouped around our three focal themes: (1) individual representations about the pandemic, which encompasses measures of coronavirus-related associations, good/bad things about the virus, and beliefs about its causes and higher-order meaning; (2) behavioral tendencies, including measures of daily activities, compliance with the health safety measures and quarantine, as well as self-efficacy beliefs and preparedness to act in the current pandemic situation; and (3) perceived impact of the pandemic, comprising measures of threat perception and the four dimensions of psychological distance toward the pandemic. We present the associational strength of those variables in relation to connectedness with nature (e.g., correlation coefficients), and report on the same associations when controlling for socially desirable responding (e.g., partial correlations). We also present ancillary analyses whenever relevant. The results below are based on our focal measure of connectedness with nature, the Extended Inclusion of Nature in Self scale, but the pattern of results is similar using the Nature Connection Index and Green Self-Identity measures as well (these additional analyses can be found in the Supplementary Material). All results are reported separately for data from each of the two waves of the study. We then provide a discussion of the findings and suggest testable propositions and areas for future research on the relation between human environmental connectedness and responses to pandemic situations. These latter are highlighted in *italics* throughout our discussion sections.

## Results

### Individual Representations

Overall, participants assessed the unprecedented pandemic situation fairly negatively, regarding both the valence of their associations and valence of their attitudes about the negative/positive aspects of the pandemic (for details see Table 2). In terms of the content generated in the word association task, the most common themes participants referred to were: general information and knowledge about the current situation (e.g., “virus,” “pandemic,” “infection,” “disease,” “Wuhan,” “worldwide”); health and safety measures (e.g., “masks,” “hand washing,” “quarantine,” “social distance”) and death (e.g., “death,” “dying,” “dead,” “killing”). Among the good things about the coronavirus, participants indicated most frequently the following themes: the reduction of negative environmental impact (e.g., air and noise pollution); social ties (e.g., being in touch with family and friends, having more time with family and children) and global community (e.g., helping each other, altruism, supporting the elderly). Among the bad things about the coronavirus, the main themes mentioned were very similar to the word association task, with the additional theme of economic issues (e.g., negative market phenomena, economic crisis, money, and job loss). Additional details about the categories can be found in the Supplementary Material. Regarding the thematic categories and association/attitude valence in wave 1, no specific relationship was found between those different measures and our focal measure of connectedness with nature (*M* = 4.89, *SD* = 1.29, Cronbach’s α = 0.88). In the data of wave 2, a positive correlation (*p* < 0.05) was found between nature connectedness and the number of nature-related associations mentioned by participants (*r*_wave_2_ = 0.10); these results remained consistent also when controlling for socially desirable responding. Additionally, a positive correlation between connectedness with nature (*M* = 4.95, *SD* = 1.26, Cronbach’s α = 0.88) and valence of attitudes about the coronavirus emerged; participants with a stronger (vs. weaker) connection to nature tended to have more positive thoughts about the coronavirus, too (for details see Table 2).

**TABLE 2:** Valence of associations, valence of attitudes about the coronavirus and self-nature connection: Descriptive statistics and correlations.

Variable	*M (SD)* (Wave 1)	*M (SD)* (Wave 2)	Correlation With Self-nature Connection (Wave 1)	Correlation With Self-nature Connection (Wave 2)
Valence of associations (overall positivity)	2.67 (1.53)	2.87 (1.60)	0.067 (0.017)	0.129** (0.056)
Valence of attitudes about the coronavirus (overall positivity)	1.75 (0.79)	1.85 (0.79)	0.057 (0.026)	0.172** (0.142**)

*p < 0.05 (2-tailed); **p < 0.01 (2-tailed). Numbers in parentheses in the last two columns indicate parameters when controlling for socially desirable responding of participants. Valence of associations was measured on a seven-point Likert scale and Valence of attitudes about the coronavirus was measured on a five-point Likert scale.

Furthermore, participants in both waves seemed to be somewhat divided on the idea that the pandemic might bear higher-order purpose and meaning, epitomizing a warning signal to us people (Table 3). However, self-nature connection correlated positively with all three items measuring these representational beliefs (for details see Table 3), possibly reflecting a more pronounced tendency to attribute transcendental meaning to the pandemic in those who felt closer to nature.

**TABLE 3:** Higher-order beliefs about the pandemic and self-nature connection: Descriptive statistics and correlations.

Variable	*M (SD)* (Wave 1)	*M (SD)* (Wave 2)	Correlation With Self-nature Connection (Wave 1)	Correlation With Self-nature Connection (Wave 2)
Coronavirus has come to tell us we are not the kings of the world.	3.69 (2.13)	3.77 (2.13)	0.203** (0.173**)	0.207** (0.150**)
Coronavirus is a punishment for mankind that has lost its way.	3.19 (1.97)	3.08 (1.98)	0.156** (0.123**)	0.162** (0.084*)
Coronavirus is a way of nature warning us to stop destroying our planet.	3.41 (2.02)	3.52 (2.03)	0.234** (0.209**)	0.247** (0.188**)

*p < 0.05 (2-tailed); **p < 0.01 (2-tailed). Numbers in parentheses in the last two columns indicate parameters when controlling for socially desirable responding of participants. All items were measured on a seven-point Likert scale.

Regarding the origin of the coronavirus, participants clearly perceived it to be more of an outcome of human activities (*M*_wave_1_ = 4.99; *M*_wave_2_ = 4.80) than of natural causes [*M*_wave_1_ = 3.27; *t*(487) = –21.40, *p* < 0.001; *M*_wave_2_ = 3.41; *t*(532) = –18.07, *p* < 0.001]. At time 1, participants with a stronger (vs. weaker) connection to nature seemed to more pronouncedly attribute natural causes to the coronavirus; this was no longer the case at time 2 (for details see Table 4). Additionally, the data revealed that stronger attribution of natural causes to the coronavirus was positively correlated to the valence of attitudes (*r*_wave_1_ = 0.20; *r*_wave_2_ = 0.27) and thoughts (*r*_wave_1_ = 0.28; *r*_wave_2_ = 0.35) regarding the coronavirus pandemic, which was not the case for human-cause attribution (all *p*’s > 0.05).

**TABLE 4:** The perceived origin of the coronavirus and self-nature connection: Descriptive statistics, reliability coefficients of relevant scales and correlations.

Variable	*M (SD)* (Wave 1)	*M (SD)* (Wave 2)	α (Wave 1)	α (Wave 2)	Correlation With Self-nature Connection (Wave 1)	Correlation With Self-nature Connection (Wave 2)
Natural cause	3.27 (1.77)	3.41 (1.77)	0.87	0.87	0.109* (0.086*)	0.050 (0.044)
Human cause	4.99 (1.69)	4.80 (1.73)	0.87	0.89	0.072 (0.063)	0.122** (0.069)

*p < 0.05 (2-tailed); **p < 0.01 (2-tailed). Numbers in parentheses in the last two columns indicate parameters when controlling for socially desirable responding of participants. All items were measured on a seven-point Likert scale.

### Behavioral Tendencies

In regard to personal reactions, it can be seen that participants reported a very high compliance with global health security measures and rated their personal self-efficacy in managing the pandemic situation fairly well (for details see Table 5). They also rated their preparedness to act as quite high. We found a positive relationship between connectedness with nature and compliance with health safety measures as well as preparedness to act in the time of the pandemic. Additionally, a positive correlation (*p* < 0.01) was found between compliance with health safety measures and the pro-environmental benefits about the pandemic mentioned by participants (*r*_wave_1_ = 0.14); at time 2, a similar result was found (*p* < 0.05; *r*_wave_2_ = 0.12).

**TABLE 5:** Self-efficacy, compliance with the safety measures, preparedness to act, and self-nature connection: Descriptive statistics, reliability coefficients of relevant scales and correlations.

Variable	*M (SD)* (Wave 1)	*M (SD)* (Wave 2)	α (Wave 1)	α (Wave 2)	Correlation With Self-nature Connection (Wave 1)	Correlation With Self-nature Connection (Wave 2)
Self-efficacy	3.65 (0.92)	3.52 (0.98)	0.82	0.86	0.047 (0.056)	0.094* (0.062)
Compliance with the safety measures	6.41 (0.84)	6.25 (0.97)	0.77	0.78	0.113* (0.112*)	0.157** (0.149**)
Preparedness to act	4.19 (0.95)	4.08 (0.96)	–	–	0.114* (0.104*)	0.128** (0.107*)

*p < 0.05 (2-tailed); **p < 0.01 (2-tailed). Numbers in parentheses in the last two columns indicate parameters when controlling for socially desirable responding of participants. Self-efficacy and Preparedness to act were measured on a five-point Likert scale. Compliance with the safety measures was measured on a seven-point Likert scale.

Notable are the findings of negative correlations between self-efficacy perceptions as well as compliance with safety measures, and participant beliefs that the pandemic represents a punishment from a higher entity or a warning signal from nature (for details, see Table 6). Yet, these seem to somewhat weaken over time.

**TABLE 6:** Higher-order beliefs about the pandemic, self-efficacy, and compliance with the safety measures: Correlations.

Variable	Self-efficacy (Wave 1)	Self-efficacy (Wave 2)	Compliance With the Safety Measures (Wave 1)	Compliance With the Safety Measures (Wave 2)
Coronavirus has come to tell us we are not the kings of the world.	–0.062 (–0.054)	0.045 (0.038)	–0.048 (–0.051)	0.037 (0.028)
Coronavirus is a punishment for mankind that has lost its way.	–0.192** (–0.187**)	–0.088* (–0.071)	–0.208** (–0.215**)	–0.072^+^ (–0.088*)
Coronavirus is a way of nature warning us to stop destroying our planet.	–0.107* (–0.102*)	0.021 (–0.087*)	–0.102* (–0.106*)	0.087* (–0.078)

^+^*p* < 0.1 (2-tailed); *p < 0.05 (2-tailed); **p < 0.01 (2-tailed). Numbers in parentheses in the last two columns indicate parameters when controlling for socially desirable responding of participants.

Regarding daily activities, respondents in general reported to watch TV, talk to relatives and friends, spend time on social media and read a lot more in the current situation than usual (for details see Table 7). A logistic regression analysis was performed to examine the association between self-nature connection and the activities people found themselves doing more than usual during the pandemic. With respect to nature-related activities such as gardening, going for a walk/hike in nature (wave 1 only), and outdoor sports, a significant relation was found. For nature-unrelated activities, a significant relation was found for some, like reading (wave 1), indoor sports (wave 2), and eating healthy.

**TABLE 7:** Activities people engage in while home and self-nature connection: Frequencies and logistic regression coefficients.

Variable	Frequency (%) (Wave 1)	Frequency (%) (Wave 2)	β (Wave 1)	β (Wave 2)
Watching movies and TV shows	84.5	79.6	0.012 (0.033)	0.017 (–0.028)
Gardening (incl. interior plants)	15.6	27.3	0.389** (0.369**)	0.234** (0.212**)
Reading	50.7	55.6	0.146** (0.138*)	0.130^+^(0.086)
Social media	66.1	65.9	–0.029 (–0.036)	0.055 (0.017)
Going for a walk/hike in nature	27.1	32.8	0.175** (0.196*)	0.094 (0.129^+^)
Sport/exercising (indoor)	26.7	32.2	0.155^+^ (0.117)	0.307** (0.274**)
Sport/exercising (outdoor)	8.8	14.0	0.230^+^ (0.256^+^)	0.188^+^ (0.239*)
Eating healthy	37.4	40.1	0.194* (0.141^+^)	0.208** (0.146*)
Talking to relatives and friends	53.8	47.9	–0.007 (–0.012)	0.020 (0.028)

^+^*p* < 0.1 (2-tailed); *p < 0.05 (2-tailed); **p < 0.01 (2-tailed). Numbers in parentheses in the last two columns indicate parameters when controlling for socially desirable responding of participants.

### Perceived Impact of the Pandemic

An important finding about the perceived threat of coronavirus (for details see Table 8) is the observable tendency to think that the pandemic and its effects do not have as much impact on oneself (*M*_wave_1_ = 4.78; *M*_wave_2_ = 4.66) as on others around us [*M*_wave_1_ = 5.34; *t*(487) = –6.76, *p* < 0.001; *M*_wave_2_ = 5.16; *t*(532) = –6.95, *p* < 0.001]. These results could indicate a potential optimistic self-bias which, in this case, represents the tendency to believe that other people are more threatened by this virus than oneself.

**TABLE 8:** Perceived threat and self-nature connection: Descriptive statistics and correlations.

Variable	*M (SD)* (Wave 1)	M *(SD)* (Wave 2)	Correlation With Self-nature Connection (Wave 1)	Correlation With Self-nature Connection (Wave 2)
The coronavirus is a threat to you personally.	4.78 (1.84)	4.66 (1.84)	0.288** (0.277**)	0.195** (0.170**)
The coronavirus is a threat to people around you.	5.34 (1.61)	5.16 (1.66)	0.211** (0.208**)	0.179** (0.177**)
The coronavirus is a threat to people in your country.	5.76 (1.39)	5.53 (1.44)	0.183** (0.194**)	0.195** (0.184**)
The coronavirus is a threat to humans in general.	5.79 (1.42)	5.64 (1.46)	0.154** (0.165**)	0.189** (0.175**)
The coronavirus is a threat to the natural environment in general.	3.45 (2.02)	3.57 (2.09)	0.125** (0.078)	0.154** (0.067)
The coronavirus is a threat to the natural environment in your country.	3.56 (2.11)	3.61 (2.10)	0.128** (0.081)	0.161** (0.077)
The coronavirus is a threat to the natural environment around you.	3.45 (2.09)	3.59 (2.12)	0.112* (0.059)	0.156* (0.069)

*p < 0.05 (2-tailed); **p < 0.01 (2-tailed). Numbers in parentheses in the last two columns indicate parameters when controlling for socially desirable responding of participants. All items were measured on a seven-point Likert scale.

A positive correlation was found between self-nature connection and perceived threat to the self and to others (Table 8). Furthermore, to gauge the relationship between the magnitude of self-bias in perceived threat and connectedness with nature, we have created a difference score by subtracting the score on personally perceived threat from the score of threat perceived for other people around. The magnitude of this difference score was negatively correlated with self-nature connection (*r*_wave_1_ = –0.167, *p* < 0.001), showing that people who felt closer to nature perceived the pandemic to threaten themselves and other people more equally. The magnitude of this difference score was no longer significantly related to self-nature connection at the second time of data collection (*r*_wave_2_ = –0.054, *p* = 0.213).

A notable finding on the more detailed psychological distance measures (for details see Table 9) is the trend to think that the pandemic and its effects are perceived as quite impactful on oneself, yet with a grain of optimistic self-bias. People for example seem to believe that others who are similar to themselves are also less affected by the pandemic (*M*_wave_1_ = 3.68; *M*_wave_2_ = 3.59) than those who are different [*M*_wave_1_ = 3.85, *t*(487) = –2.78, *p* < 0.001; *M*_wave_2_ = 3.85; *t*(532) = –5.35, *p* < 0.001]. They consider their own lives to be affected by the pandemic later in time (*M*_wave_1_ = 5.84; *M*_wave_2_ = 5.70) than the lives of other people, even when they reside in the same area [*M*_wave_1_ = 6.11, *t*(487) = –5.88, *p* < 0.001; *M*_wave_2_ = 6.03, *t*(532) = –4.58, *p* < 0.001]. Most relevantly, people regard their own chance of becoming infected with the virus (*M*_wave_1_ = 41.45%; *M*_wave_2_ = 38.57%) to be lower by around 18% in comparison to how likely this can happen to other people from the same area [*M*_wave_1_ = 59.72%; *t*(487) = –16.44, *p* < 0.001; *M*_wave_2_ = 55.97%; *t*(532) = –13.86, *p* < 0.001]. We observe this trend despite the fact that participants see their own area to be more affected by the coronavirus pandemic (*M*_wave_1_ = 4.03; *M*_wave_2_ = 3.91) than areas that are geographically far away [*M*_wave_1_ = 2.94; *t*(487) = 12.44, *p* < 0.001; *M*_wave_2_ = 3.02; *t*(532) = 19.33, *p* < 0.001].

**TABLE 9:** Psychological distance and self-nature connection: Descriptive statistics and correlations.

Variable	*M* (SD) (Wave 1)	*M* (SD) (Wave 2)	Correlation With Self-nature Connection (Wave 1)	Correlation With Self-nature Connection (Wave 2)
My local area where I live is likely to be affected by the coronavirus pandemic.	4.03 (1.04)	3.91 (1.06)	0.059 (0.077)	0.107* (0.158**)
The coronavirus pandemic will mostly affect areas that are far away from where I live.	2.94 (1.50)	3.02 (1.44)	0.060 (0.037)	0.039 (0.009)
The coronavirus pandemic is likely to have a big impact on people like me.	3.68 (1.12)	3.59 (1.12)	0.172** (0.173**)	0.204** (0.203**)
People very different from me are going to be greatly affected by the coronavirus pandemic.	3.85 (1.09)	3.85 (1.04)	0.091* (0.095*)	0.127** (0.160**)
Please indicate how familiar you are with the closest person in your environment that is directly affected by the coronavirus.	2.56 (1.44)	2.81 (1.46)	0.123** (0.107*)	0.151** (0.145**)
When will the coronavirus pandemic start affecting your personal life?	5.84 (1.62)	5.70 (1.68)	0.076 (0.088)	0.149** (0.202**)
When will the coronavirus pandemic start affecting the lives of people in your area?	6.11 (1.35)	6.03 (1.42)	0.043 (0.065)	0.168** (0.219**)
What do you think is the likelihood (in%) that you personally will contract the coronavirus?	41.45 (29.14)	38.57 (28.98)	0.151** (0.140**)	0.117** (0.112*)
What do you think is the likelihood (in %) that people in your area will contract the coronavirus?	59.72 (31.08)	55.97 (30.27)	0.104* (0.113*)	0.084 (0.100*)

*p < 0.05 (2-tailed); **p < 0.01 (2-tailed). Numbers in parentheses in the last two columns indicate parameters when controlling for socially desirable responding of participants. Geographical and social distance were measured on a five-point Likert scale. Temporal distance was measured on a seven-point Likert scale. Hypothetical distance was measured as percentage out of 100.

With regards to connectedness with nature, the correlations with measures of perceived social and hypothetical distance toward the pandemic show a significant association (Table 9). More specifically, those who report being more connected to nature also report higher experience of the pandemic’s impact on oneself and others, and greater personal knowledge of people who are infected by the virus. They also estimate a higher likelihood of contracting the virus themselves and a higher likelihood of others in their area contracting it as well, further pointing to a possible reduction of self-bias for strong (vs. weak) self-nature connection individuals (Table 9). In a similar vein, people with stronger self-nature connection reported to feel more familiar with the infected people around them.

Furthermore, several of the measures reported in Table 9 also significantly correlate (*p* < 0.05) with the health security compliance scores reported in Table 5. Specifically, people who perceive the pandemic to increasingly affect people, both similar to them (*r*_wave_1_ = 0.25; *r*_wave_2_ = 0.25) and different (*r*_wave_1_ = 0.17; *r*_wave_2_ = 0.19), and who perceive the onset of the pandemic’s impact as current rather than late, both for themselves (*r*_wave_1_ = 0.39; *r*_wave_2_ = 0.29) and for other people in their area (*r*_wave_1_ = 0.43; *r*_wave_2_ = 0.32), reported higher adherence to the health security regulations. Similarly, the higher the perceived impact of the pandemic on the area where one lives (*r*_wave_1_ = 0.28; *r*_wave_2_ = 0.29), the higher the reported compliant behavior. Perceiving areas far away to be greatly impacted by the virus is, however, negatively correlated with compliant behavior (*r_wave_1_* = –0.11; *r*_wave_2_ = –0.10). Interestingly, increased perceived likelihood of actually contracting the virus oneself is not associated with compliant behavior, but the perception that other people in the surroundings might is (*r*_wave_1_ = 0.14; *r*_wave_2_ = 0.17). All these results remain consistent also when controlling for socially desirable responding.

## Discussion

### Individual Representations

In both waves of data collection, our participants expressed a grim view of the current pandemic, appropriate to the gravity of the situation, and did not seem to take it lightly. Based on their most frequent associations and the aspects of the pandemic mentioned most, they have a detailed knowledge about the situation, along with the seriousness or even fatality of the risks the coronavirus brings along. People seem to be conscious of the inevitable negative economic consequences caused by the pandemic, as millions of United States citizens have filed for unemployment since the pandemic’s outbreak (Bell and Blanchflower, 2020; Marte and Sullivan, 2020). However, when prompted, participants also associated the crisis with some salient positive aspects; mainly relating to alleged environmental benefits and enhancement of social ties. At an early stage (wave 1), these knowledge structures did not relate to participants’ level of connectedness with nature and might have reflected the ubiquitous availability of coronavirus-related information on public/social media channels. Overall, while participants seemed well-informed about the nature of the pandemic, they initially largely reported globally attended views on it. Therefore, *it remains to be seen in future studies whether the initially acquired knowledge and perceptions about a pandemic situation reflect the deeply held personal convictions (vs. stereotypical knowledge) for individuals who are more strongly (vs. weakly) connected with nature, and to what extent those cognitions are differentially predictive of behaviors in pandemic-like situations.*

In wave 1 of data collection, the strength of individual self-nature connection was not related to how positive/negative an individual perceived the critical situation nor to particular categories of mental associations. However, a month later, the results of the second data collection showed a more positive perception of the pandemic for individuals with a stronger (vs. weaker) connectedness with nature, as well as a positive relationship between the latter and the number of spontaneous nature-related associations mentioned. This shift between the two data collections might be explained by the initial novelty and subsequent adjustment to the phenomenon. Indeed, the data shows a slight increase in perceived positivity of the pandemic from time 1 to time 2, and significantly so for people with a stronger self-nature connection. One possibility is that over time, stronger (vs. weaker) nature-connected individuals attend to, and value more, the positive aspects of the pandemic such as its environmental benefits. Research in identity theory indeed suggests that people with a stronger (vs. weaker) specific identity tend to consider more the pieces of information in their broader environment that are relevant and supportive of that identity (Reed et al., 2012). *Future research should therefore systematically investigate whether individuals with a stronger (vs. weaker) nature connectedness gradually internalize more positive information about pandemic crises and whether identity motivations underlie these information processing strategies.*

Addressing our initial speculation about the link between individuals’ perceptions of the coronavirus origins and the extent of their overall positive/negative outlook on the pandemic, we find that in general, attribution of natural causes to the pandemic was associated with more positive thoughts and attitudes about the latter. However, at time 1, a higher (vs. lower) nature connectedness was associated with a stronger attribution to natural causes without being accompanied by more positive thoughts/attitudes about the pandemic. At time 2, a higher (vs. lower) nature connectedness was associated with a more positive view of the pandemic, despite the lack of its stronger attribution to natural causes. This data suggests a potentially more nuanced attitude formation process for individuals with a stronger (vs. weaker) nature connectedness. *A relevant question for future research is to investigate whether the strength of individual self-nature connection determines not only how new knowledge structures and attitudes form initially but also how they develop over time. It is also important to consider how the role attribution to nature vs. humans in the development of a pandemic is linked to the formation of attitudes, and why and how this attitude formation process is potentially more complex for people with a stronger (vs. weaker) connectedness with nature. Conversely, further investigation could also determine whether the progressive acquisition of new knowledge structures about pandemics, including knowledge about its potential causes, can influence (positively or negatively) a person’s connectedness with nature over time.*

Across both waves of data collection, we observe a positive relation between self-nature connection and people’s tendency to view the pandemic as a warning signal from a higher entity, whether ambiguous or from nature specifically. *Does this reflect a higher level of transcendental beliefs in strong (vs. weak) self-nature connection people in the case of a new and unknown pandemic situation?* Religious beliefs can help one to cope with increased salience of negative life events (Pargament, 1997), and future research might wish to test the same regarding beliefs about nature as a higher-power entity. In that regard, *it would be important to find out whether transcendental nature-related beliefs could offer similar functions as some spiritual or religious beliefs in times of crisis, and whether these functions become salient for strongly (vs. weakly) nature-connected people in pandemic-like situations*. Such investigations could follow in the footsteps of the long-standing premises that religious belief systems have substantial implications for other contents and properties of the self-concept (Blaine et al., 1998; Vess et al., 2012).

### Behavioral Tendencies

There are high reports of compliance with the recommended health and safety measures at both times of data collection; generally showing a great individual readiness to adopt new behaviors durably in the face of a global danger. Interestingly, this tendency seems to be associated with a stronger connectedness with nature. This increased compliance might reflect the idea that people with a more pronounced connectedness with nature want to remain protective of nature, including themselves and all living beings (Clayton, 2003). While there was a positive connection between compliance with health safety measures and the pro-environmental benefits about the pandemic mentioned by participants in both waves, the mentions of these benefits were significantly higher for people with stronger (vs. weaker) self-nature connection only in the second wave of data collection, hinting to a more complex processes underlying the connection between compliance and self-nature connection. Relatedly, it could also be that people with a stronger (vs. weaker) self-nature connection are, to a certain extent, more prepared to adopt necessary changes in a given situation because they are more used to protecting the broader natural environment and the planet in general (Mackay and Schmitt, 2019). They are also more likely to cooperate with others to achieve an important common goal (Zelenski et al., 2015). In that sense, they have prior experience in taking concrete steps to mitigate behaviors with negative global impact and the effort to adopt new forms of conservational behavior may not be that high for them. A general support for this idea might be offered by the experience sampling account of attitude formation (Fiedler, 2000); suggesting that peoples’ experiences of the world are limited and selective and therefore, their subsequent tendencies might be biased in favor of their earlier experiences. In other words, current attitudes and related behaviors are reflective of previous experiences and are mutually reinforced. Consequently, people who have fewer experiences with engagement in conservational behaviors might have more difficulty with the enactment of other, related behaviors, such as the habit-changing and preventive behaviors recommended to adopt in the global fight against the current pandemic. In line with this idea, a research proposition might be that *in times of a pandemic, people with a stronger (vs. weaker) connection to nature tend to be more prepared and/or require less effort to adopt the behavioral changes needed to cope with the situation, and will therefore be able to respond to it better and/or possibly faster.* Related research might focus on whether *the stronger (vs. weaker) nature-connected people’s more frequent engagement in pro-environmental behaviors acts as a mediator in their tendency to adopt the new preventive behaviors.* It would also be important to find out more about *how the pandemic experience and adoption of relevant preventive behaviors would impact the adoption of future pro-environmental behaviors and whether those tendencies may boost one’s sense of connectedness with nature.*

Furthermore, the present data suggests that people with a strong connection to nature tend to perform activities that are nature-related more than usual, such as gardening or outdoor sports, possibly helping them to gain benefits from the restorative power of natural spaces (Hartig et al., 2014; Collado et al., 2017) even when restrictions in external environment apply. *Future research might therefore investigate whether a stronger (vs. weaker) connection to nature might lead to the enactment of particular coping strategies (e.g., activities that uphold this connection) that could be globally helpful in dealing with and adjusting over time to novel pandemic-like situations.*

On the other hand, nature-related activities can also be seen as a potential health hazard in pandemic times if these are performed outside of the home and infringe on the confinement regulations. For example, people with stronger self-nature connection reported at an earlier time point in the pandemic (wave 1) to go more out for walks and hikes in nature. In this sense, future research could study the proposition of limited behavior compliance, that *initially, people with a strong (vs. weak) self-nature connection might comply with government-imposed security measures, but only to a point. If the safety regulations imposed prevent them from expressing their attachment to nature (e.g., walking in nature), at first they might consider infringing on these regulations, and this could be a potential danger to mitigate a pandemic in its early stages. Further investigations may also test whether the motivation underlying this trade-off is related to the expected restorative, or even reinvigorating, capacity of unspoiled natural environments (Hartig et al., 2014).* Yet, the comparison between wave 1 and wave 2 shows that over time, individuals with a stronger (vs. weaker) sense of nature connectedness reported to do more frequently only those nature-related activities that could be done at home, thus being more compliant with the movement-restricting health and safety measures.

Moreover, people with a strong connection to nature also tend to engage more in healthy eating, reading, and indoor sports. The reports of healthier eating and indoor sports might reflect previous empirical findings showing that some aspects of sustainable consumption carry a health halo (e.g., organic food is perceived as healthier than conventional food, Sundar and Kardes, 2015). The link to increased reading and indoor sports raises speculations about *which activities, not directly involving nature, may be beneficial for the adaptable maintenance, or perhaps even the enhancement, of self-nature connection and why.*

We further find a negative relation between some of the higher-power beliefs about the pandemic, perceived self-efficacy, and behavior compliance with safety measures, especially in the initial stage of the pandemic (Table 6). These trends may reveal a potential danger—beliefs about the pandemic being a message from a higher-power entity might lead to the initial reaction to the pandemic conveying general individual inaction and the conviction that one’s actions have no power to switch the course of events. Taking into account that these higher-power beliefs are stronger for people with stronger self-nature connection (Table 3), which in turn relates positively to compliance with safety measures and self-efficacy (Table 5), our exploratory data offers an intriguing pattern of results. Previous research showed that intrinsic religious beliefs might lead to an increase in perceived self-efficacy when faced with a potentially dangerous event (e.g., terrorist attack; Fischer et al., 2006). Specifically, in cases where people experience low self-efficacy, reminders of external forces that ensure contingency in the world seem to help them to circumvent their own feelings of powerlessness and commit stronger to their goals (Khenfer et al., 2017). While remarking that the measures of transcendental higher-entity beliefs pertaining to the meaning and purpose of the pandemic do not gauge any particular belief system, the mentioned related empirical findings are inspirational. *Especially in regard to initial experiences of a novel pandemic-like situation, it could be proposed that higher-power beliefs about nature (i.e., nature being a higher force that gives meaning and consistency to the world) might be one correlate of nature connectedness that, in cases of novel behavioral expectations, acts as a facilitator helping to transform relevant motivations into future required behaviors.*

### Perceived Impact of the Pandemic

Taken together, the present data suggests that people in general perceive the magnitude of the pandemic’s exertion on their own lives to be significantly lower than on other peoples’ lives. This finding appears to be in line with empirical research showing that people underestimate their own risk for aversive life events (Greening and Chandler, 1997) and in line with the body of research on the cognitive bias known as “unrealistic optimism” found for perceptions of environmental and technological risks (Costa-Font et al., 2009). This effect has also been shown with other types of diseases such as HIV, where people underestimate the risk of contracting the virus themselves (Ijadunola et al., 2007). The same underestimation was reported during the 2003 SARS outbreak in Hong Kong (Tang and Wong, 2003; Manuell and Cukor, 2011). While our data does not indicate whether this asymmetry in risk prediction represents a risk-underestimation for self or a risk-overestimation for others, it looks like this asymmetry also applies to the novel situation of the currently ongoing pandemic. This might be a potentially dangerous mindset, as it amounts to underrating individual potential of actually contracting a virus whose real infection rates remain largely unknown and might lead to individual failure to take preventive actions (Weinstein et al., 1990).

Those who feel the impact of the pandemic on their own and others’ lives as being greater reflected the expectation to be more likely to contract the virus. Based on the first wave of data collection and on the result that people who feel closer to nature perceive the pandemic to threaten themselves and other people in a similar fashion, *one future area of research could be the study of the positive impact of self-nature connection on reducing self-bias in pandemic risk estimations when the situation is still very new and unknown.* We also suggest that *this process may be mediated by strongly (vs. weakly) nature-connected people nurturing a special type of social identity, whereby they would tend to view themselves and other humans as part of a global “nature” in-group that comprises all other living forms as well.* On the other hand, it is worth investigating *whether accepting that oneself is also at risk, just like other humans, by a presumably natural event might in turn reinforce individuals’ connection to the natural environment.*

Relatedly, it is plausible that thinking of nature as a substantial part of oneself might help one realize the magnitude and immediacy of nature-related dangers at hand more easily. A potential question for future research is *whether a closer connection to nature might indeed facilitate more realistic (or overestimated) perceptions about the consequences of natural events for people’s lives*. Another valuable area for future inquiry is to test *whether experiencing large disturbances in one’s life due to events that originate (vs. those that do not) in the natural environment could change people’s connection to nature in a more (vs. less) persistent manner.*

Our data also suggests that the more the pandemic affects people’s lives psychologically, the higher their compliance with the recommended health security measures needed to reach global control over the disease. Both psychological perception of the pandemic’s impact and compliance tendency are more pronounced for people with a stronger (vs. weaker) self-nature connection, as indicated by our results. Relatedly, future research might examine *whether increased compliance with protective measures during a pandemic reported by individuals with stronger self-nature connection is mediated by their feeling of being potentially also more affected by the virus due to their more inclusive position in the natural environment.* Furthermore, do these compliance tendencies reflect an egoistic concern, whereby the perception of lack of control over others’ actions prompts intentions to engage more in personal actions that have health benefits? Or may they also signal a genuine altruistic motivation in the form of care for others? We suggest that future research is merited to investigate *whether more selfish vs. more altruistic motivations underlie the relationships between self-nature connection, perceived psychological distance of a pandemic, and compliance with societal safety norms.*

## Conclusion

The coronavirus pandemic has brought about an unprecedented challenge for humankind at a global level. Medical research has been very active to find out more about the pandemic and identified its origins in complex nature-human interactions (Di Marco et al., 2020; Zhou et al., 2020). Environmental scientists also need to better understand the psychological implications of those interactions at the individual level.

The present research contributes to this important effort with a two-wave exploratory survey focusing on individual psychological reactions in pandemic times. Taking our exploratory results as a case in point, we have put forward a series of research questions and propositions that can be systematically tested in future investigations. Our exploratory findings suggest that connectedness with nature, as an important individual identity trait, may consistently shape individual reactions to, and in turn be influenced by, global pandemic crises, both in the initial stages of the latter and over time. The overall picture emerging from our study is one that depicts complex and partially time-dependent, yet often positive relations between connectedness with nature, individual representations, behavioral tendencies, and perceived impact of the pandemic on one’s life and the lives of other humans. Regarding individual representations, our data suggests that self-nature connection may be related to a specific understanding of where the pandemic comes from and to specific beliefs about its origins. Concerning behavioral tendencies, our findings indicate links between nature connectedness and the behaviors people engage in during pandemics, specifically their compliance with health and safety measures as well as daily enactment of nature-related and nature-unrelated activities. In terms of perceived impact, the results suggest that self-nature connection is associated to views on whether the pandemic represents a psychologically close or distant threat to oneself or others.

Future research themes might focus on clarifying the cognitive structures related to self-nature connection, specifically targeting those that are activated by pandemic-like situations and have the potential to act as either strong motivators or strong barriers of individuals’ actions, initially and over the time of adjustment. Such research efforts might also want to target the role of self-nature connection in knowledge acquisition, information processing and attitude formation, specifically in the emerging and hazardous context of believing and spreading of misinformation, unscientific knowledge, and hoaxes. Research attention ought to be placed on examining the part self-nature connection plays in people’s perception of threat, estimation of risks and the use of cognitive heuristics in health-hazardous situations. Future investigations may also focus on the significant aspects of the experiential overlap between self-nature connection, its related sustainable behavioral tendencies and preventive health and safety actions.

Taken together, our exploratory findings are suggestive of the conclusion that the connection to the natural environment individuals develop throughout their lives represents a valuable construct, and a steppingstone to further our understanding about individual behaviors in global crisis situations originating in human-nature interactions. Yet, the present findings also hint to the possibility that self-nature connection at its core can be associated with complex time-dependent psychological mechanisms such as altruistic and selfish actions, personal knowledge and stereotypical cognitions, as well as preparedness to act and blissful inactivity. This warrants further in-depth investigations along the lines of the research propositions and directions advanced in this paper.

Nevertheless, we urge readers to take a note of the exploratory nature of our findings. Although exploratory studies have been “remarkably underutilized” in extant research (Stebbins, 2001, p. 3), there is a general agreement that this type of initial investigation is appropriate to address novel and/or under-researched questions and can serve as a platform for future investigations, refinement of research questions, as well as hypothesis and theory development (Stebbins, 2001; Creswell, 2009; Sarantakos, 2013). While a good deal of exploratory research employs a qualitative approach (Stebbins, 2001; Creswell, 2009), we opted for a mostly quantitative study for reasons of availability of relevant published measures regarding the majority of constructs we wished to investigate. Our exploratory study addressed a relatively open research question about the relations between individuals’ self-nature connection, perceptions of, and reactions to a global epidemic. The reported and discussed results largely rely on associational evidence. In addition, the relationships that have emerged are characterized by small to medium effect sizes, thus readers ought to exercise caution when interpreting our results from the perspective of theoretical frameworks and causal models. Our interpretations based on the exploratory data are tentative; our statistical analyses aimed at discovery rather than validation, and offer preliminary insights along with propositions that require to be systematically studied in subsequent hypotheses-testing research. To that effect, our study might also provide valuable insights by informing future research investigating these important issues on the choice of materials, methodologies, and sample size estimations.

It is also critical to be mindful of the contextual variables and limitations that were not addressed by the present study, yet likely contributed to shaping the global situation and individual reactions during the rapidly changing pandemic times. Indeed, our study was conducted using an online platform and we hired United States residents to complete the two waves of our survey. Though our sample performed fairly well when we compared its demographic characteristics with the official census data, by no means can we claim that it was fully representative of the current United States population. Moreover, the United States is a multicultural country with a wide array of social, ethnic, and religious diversity and these socio-cultural variables might systematically factor in into our findings.

In the literature, it has been found that culturally dependent aspects such as spirituality are positively related to nature connectedness (Kaza, 1993; Kamitsis and Francis, 2013). Scholars have additionally considered other cultural factors, such as ethnicity, to identify possible differences in nature representations and connectedness with nature (Mayer and Frantz, 2004; Taylor, 2019). While the assessment of such cultural variables was beyond the scope of the present paper, future research might remedy this limitation by identifying and evaluating the impact of relevant socio-cultural variables on nature connectedness and psychological responses to pandemics.

Although our results seemed mostly consistent over the two time points of measurement, shifts in our findings related to temporal changes could be observed. The mean levels of some variables (e.g., perceived psychological distance, representations and attitudes toward the pandemic) and their associations with self-nature connection (e.g., positive association of connectedness to nature and valence of representations about the pandemic) fluctuated between the two time points. Besides the mechanisms of psychological adjustment to a novel situation, these shifts might be partially attributed to social, political, and economic events that accompanied the development of the coronavirus pandemic in the United States. As such, the pandemic caused observable increases in deaths in March and April in the country (Fox, 2020). The economy was curtailed by the pandemic during March and April 2020 to such an extent that in these two months combined, non-farm employment rate fell by a total of 22 million (Bureau of Labor Statistics, 2020) and there was a simultaneous dramatic rise in public debt (Duffin, 2020). Besides economic struggles, individuals’ communication and information acquisition strategies had also been forced to adapt to the situation—in early March already there was an increase by 18% in United States year-on-year home-data usage, as social and professional activities had moved online (Clement, 2020). At the same time, American customers showed a significant surge of interest for online communication tools (Clement, 2020). Crucially, by heavily impacting the disadvantaged, the pandemic greatly deepened and highlighted the consequences of social and economic inequalities in the United States population (Fisher and Bubola, 2020; North, 2020). All these developments have profoundly impacted people’s perceptions, attitudes and behaviors during the pandemic and as such, might have affected our findings.

Notwithstanding the aforementioned limitations, the main objective of our exploratory investigation was to spark the interest of environmental researchers in the study of psychological responses to a pandemic by proposing a roadmap for future inquiry in this crucial and timely substantive area. We are hopeful that our research has reached its objective.

## Data Availability Statement

The datasets presented in this study are publicly available and can be found in an online repository. The concrete accession links can be found in the article’s Materials and Methods section.

## Ethics Statement

The studies involving human participants were reviewed and approved by the Ethics Committee of the Faculty of Business and Economics, University of Lausanne. The patients/participants provided their written informed consent to participate in this study.

## Author Contributions

SH, SC, and LR contributed to the conception and design of the study and wrote the first draft of the manuscript. SH, SC, LR, and NM conducted the study together and wrote sections of the manuscript. LR and SH performed the statistical analysis. All authors contributed to manuscript revision, read, and approved the submitted version.

## Conflict of Interest

The authors declare that the research was conducted in the absence of any commercial or financial relationships that could be construed as a potential conflict of interest.
