# Supplementary material for: Connectedness With Nature and Individual Responses to a Pandemic: An Exploratory Study
Source: Front Psychol. 2020 Sep 23;11:2215. doi: 10.3389/fpsyg.2020.02215 (PMC7538508; doi:10.3389/fpsyg.2020.02215)
Supplement: Supplementary file 1 [file Table_1.DOCX]

Supplementary Material

**Figure 1**

*Valence of associations, valence of attitudes about the coronavirus and self-nature connection: Scatterplot*

**
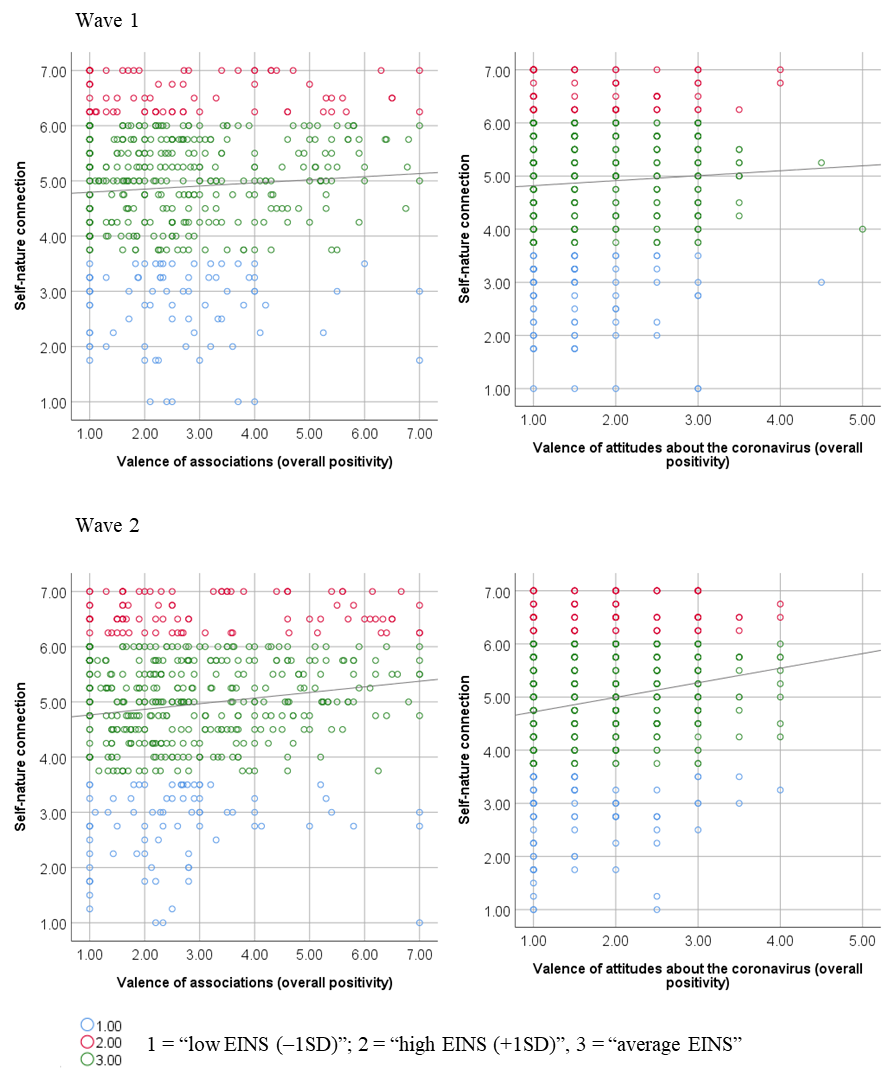
**

Note. “EINS” stands for score on the Extended Inclusion of Nature in Self scale (Martin & Czellar, 2016) – a measure of the self-nature connection.

**Figure 2**

*Higher-order beliefs about the pandemic and self-nature connection: Scatterplot*

**
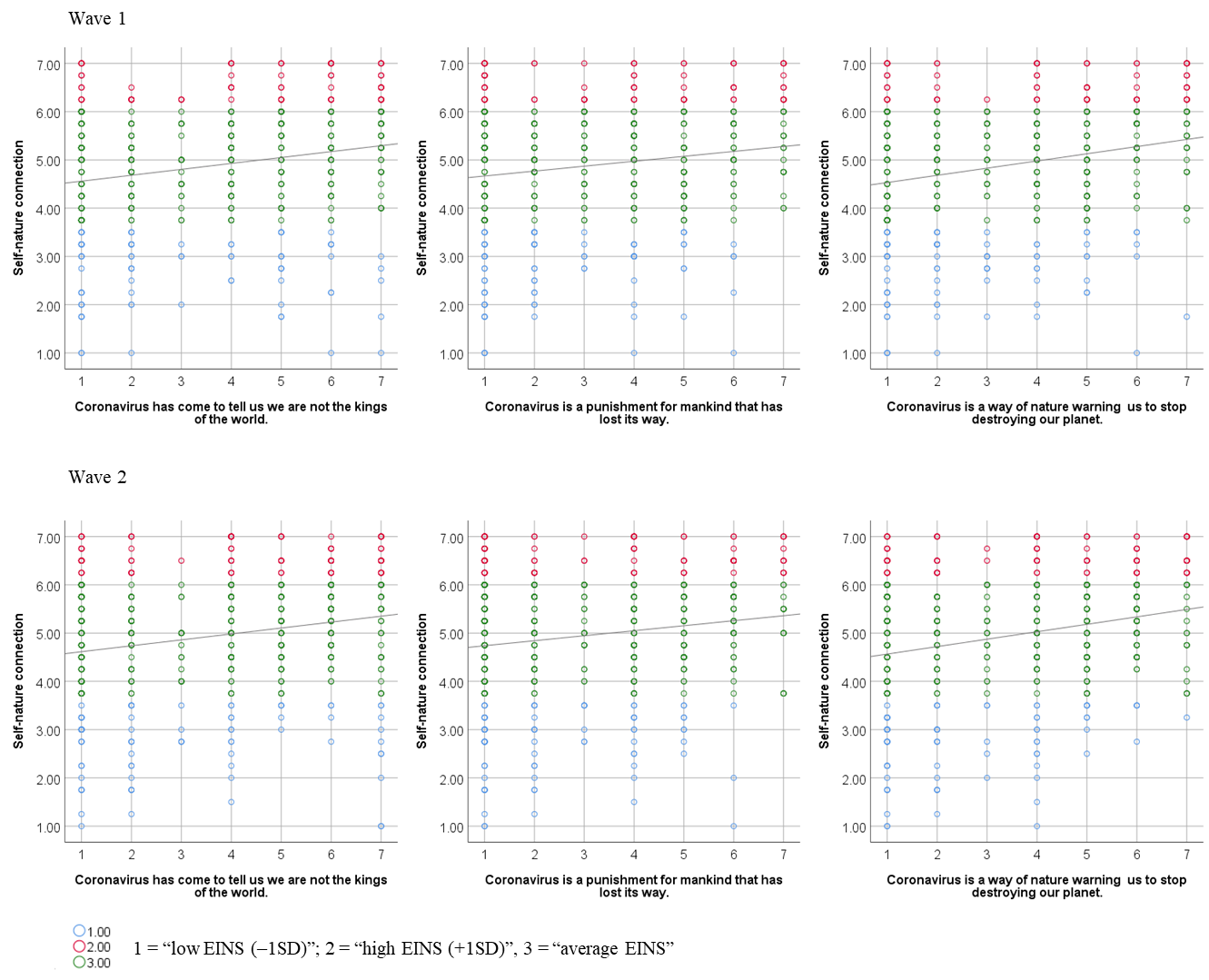
**

**Figure 3**

*The perceived origin of the coronavirus and self-nature connection: Scatterplot*

**
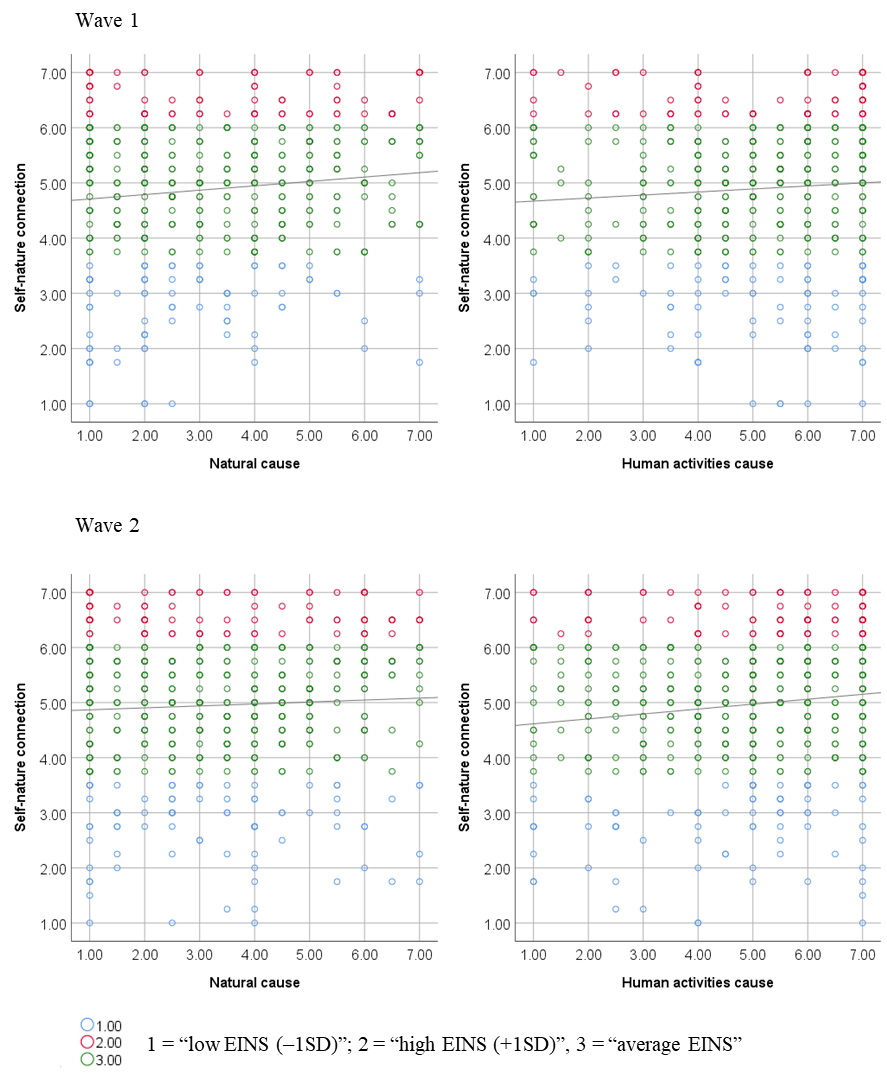
**

**Figure 4**

*Self-efficacy, compliance with the safety measures, preparedness to act and self-nature connection: Scatterplot*

**
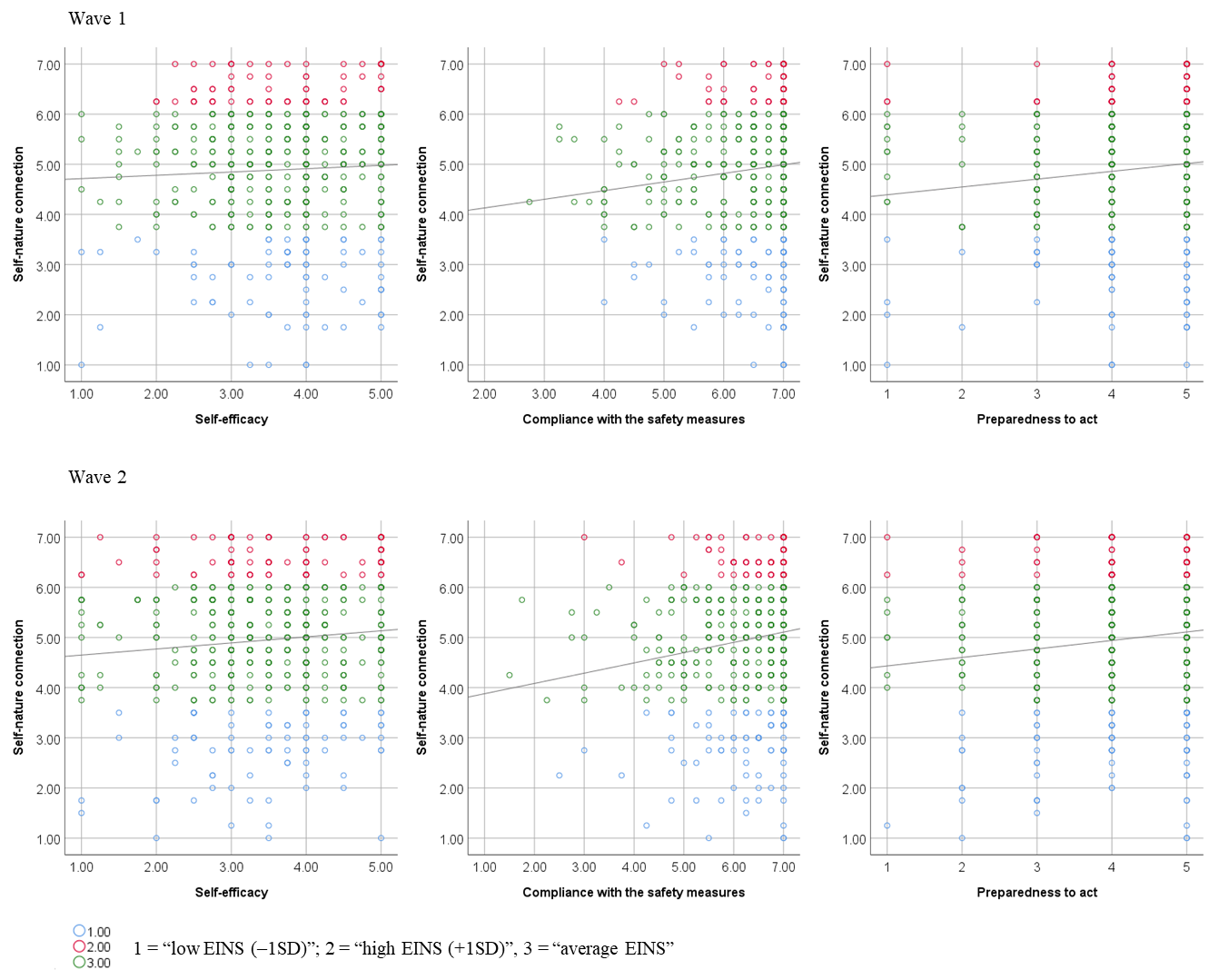
**

**Figure 5**

*Perceived threat and self-nature connection: Scatterplot*

**
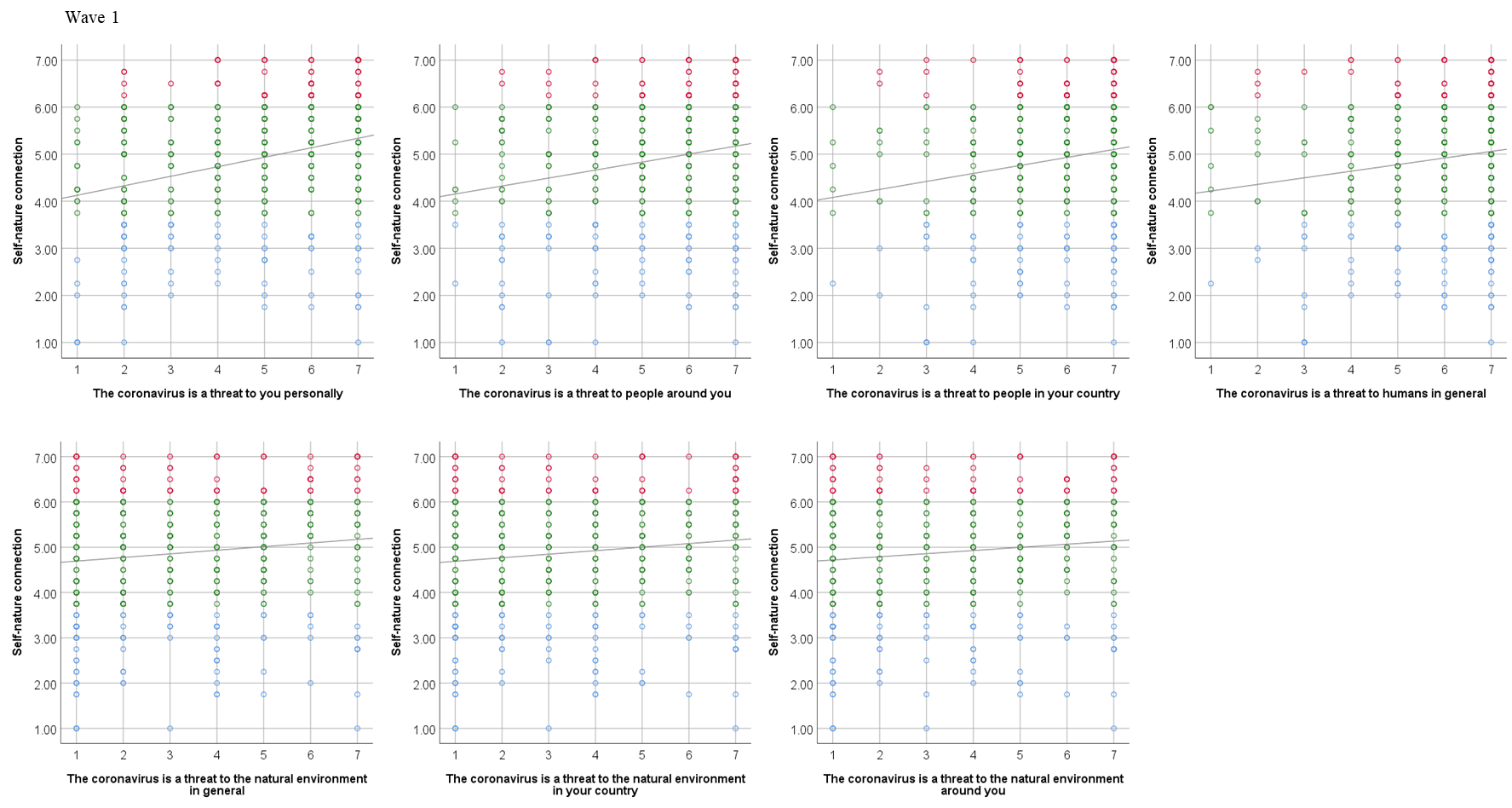
**

**
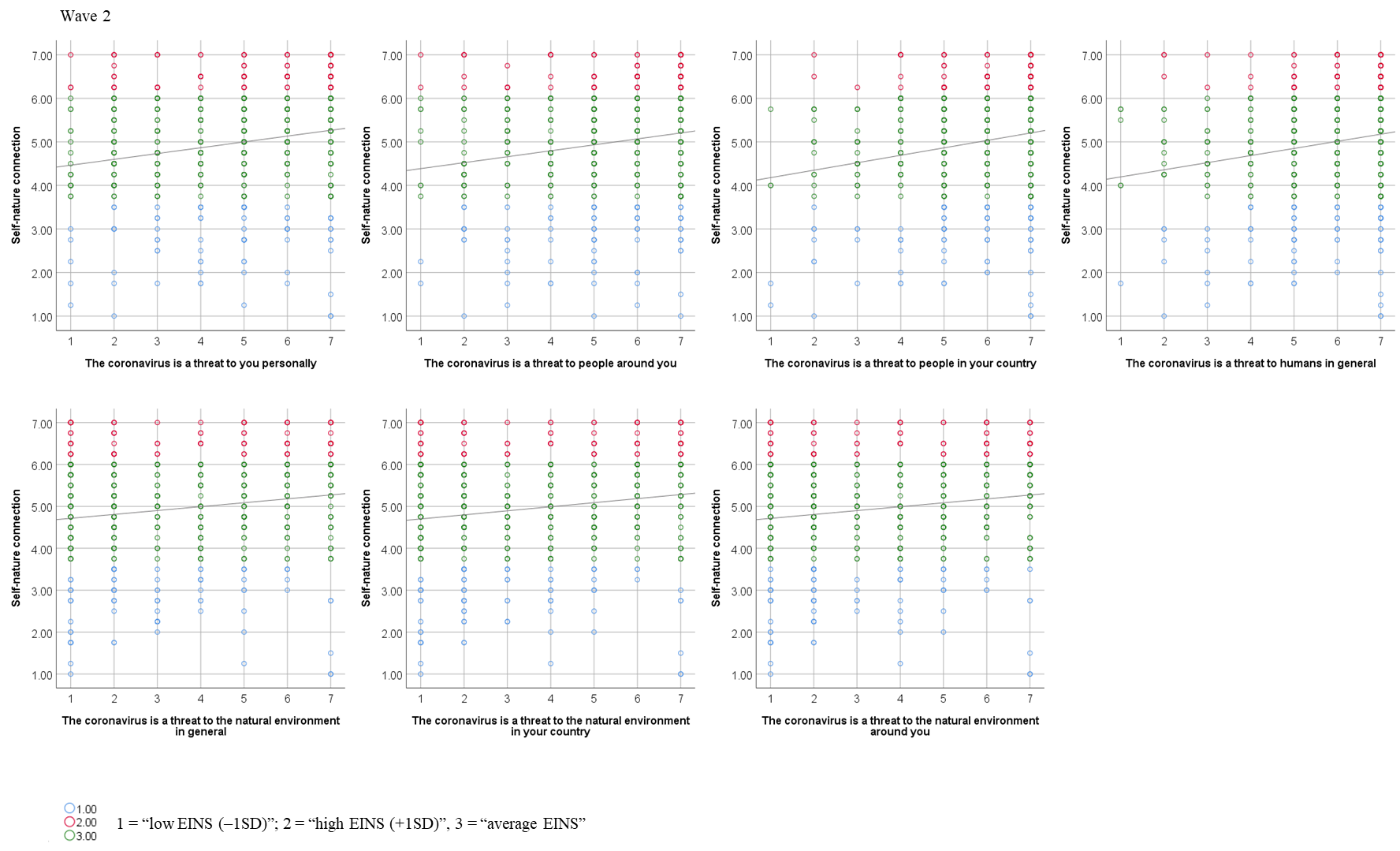
**

**Figure 6**

*Psychological distance and self-nature connection: Scatterplot*

**
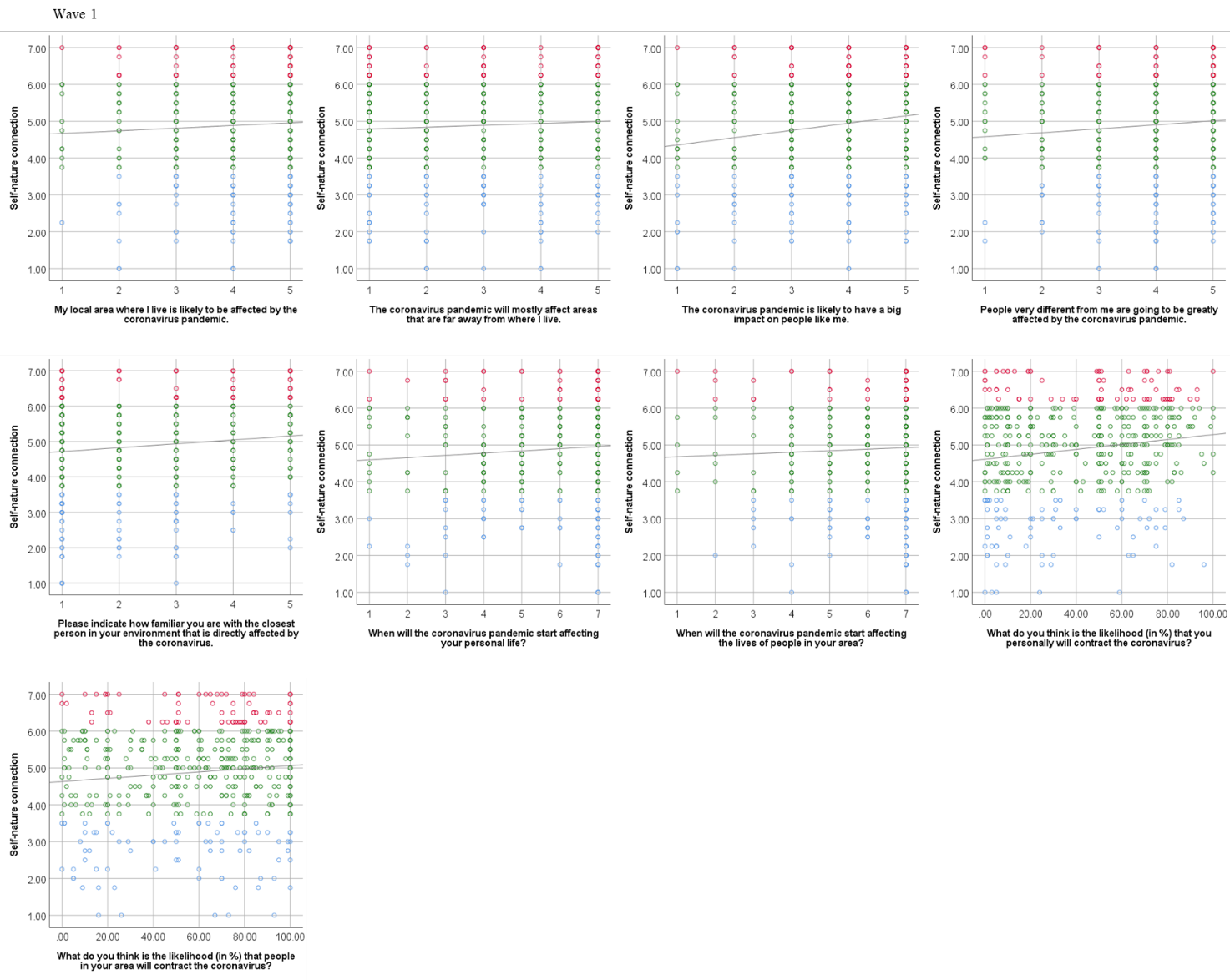
**

**
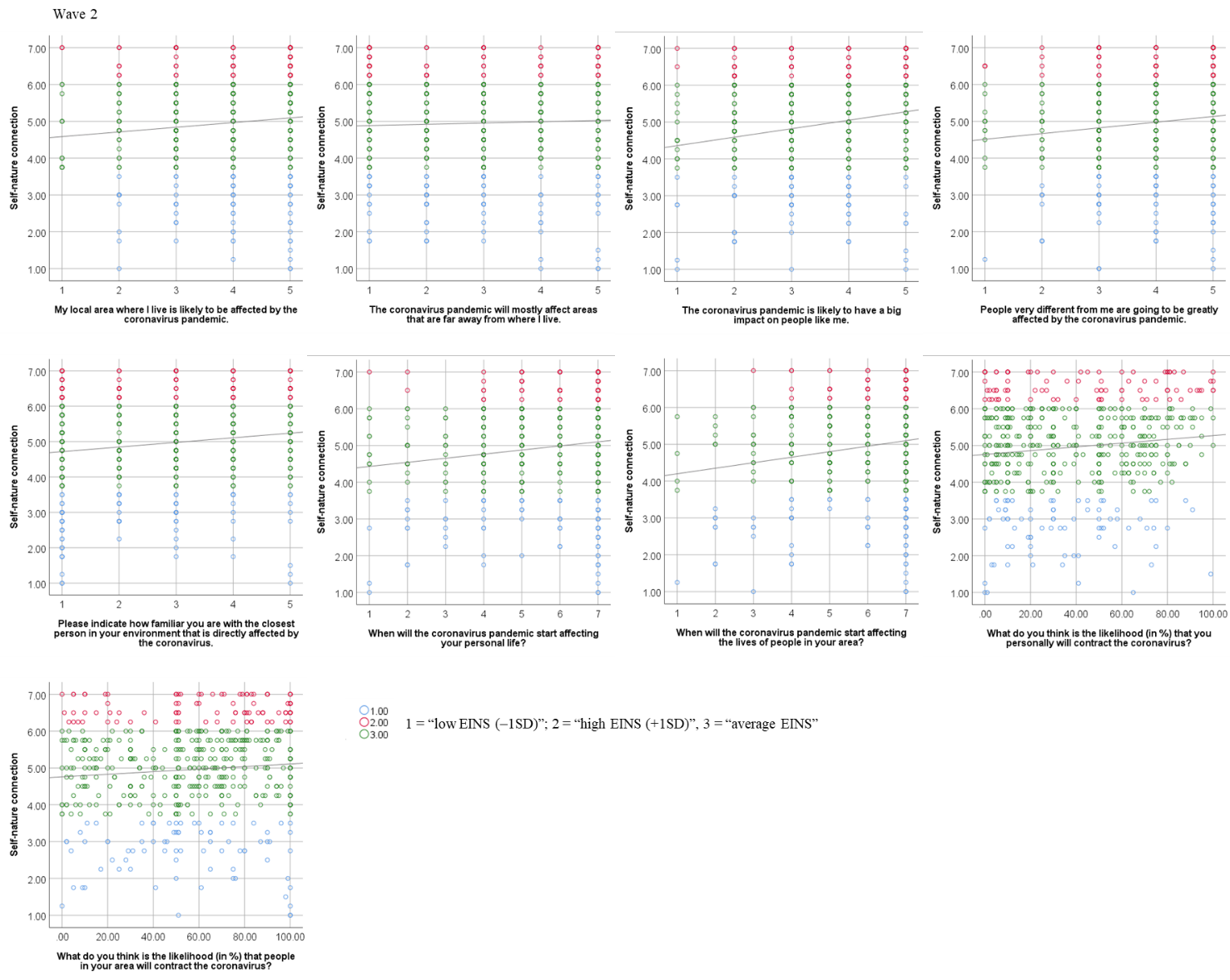
**

**Table 10a (wave 1)**

*Pearson´s correlations between measures that assess individual’s relationship with nature*

|  | Variable | M | SD | 1 | 2 | 3 |
| --- | --- | --- | --- | --- | --- | --- |
| 1. | Connectedness with Nature | 4.89 | 1.29 | 1 |  |  |
| 2. | Nature connection index | 7.22 | 1.56 | .708** | 1 |  |
| 3. | Green self-identity | 4.14 | 1.22 | .563** | .528** | 1 |

*Note. +p <* 0.1 (2-tailed); *** *p* < 0.05 (2-tailed); ** *p* < 0.01 (2-tailed). Connectedness with Nature and green self-identity were measured on a seven-point Likert scale. Nature connection index was measured on a nine-point Likert scale.

**Table 10b (wave 2)**

*Pearson´s correlations between measures that assess individual’s relationship with nature*

|  | Variable | M | SD | 1 | 2 | 3 |
| --- | --- | --- | --- | --- | --- | --- |
| 1. | Connectedness with Nature | 4.95 | 1.26 | 1 |  |  |
| 2. | Nature connection index | 7.41 | 1.41 | .657** | 1 |  |
| 3. | Green self-identity | 3.42 | 1.10 | .574** | .465** | 1 |

*Note.* ^+^*p* < 0.1 (2-tailed); *** *p* < 0.05 (2-tailed); ** *p* < 0.01 (2-tailed). Connectedness with Nature and green self-identity were measured on a seven-point Likert scale. Nature connection index was measured on a nine-point Likert scale.

Individual Representations

**Table 11**

*Valence of associations, valence of attitudes about the coronavirus and nature connection index: Descriptive statistics and correlations*

| Variable |  | |  | |
| --- | --- | --- | --- | --- |
|  | *M (SD)*  *(Wave 1)* | *M (SD)*  *(Wave 2)* | *Correlation with nature connection index (Wave 1)* | *Correlation with nature connection index (Wave 2)* |
| Valence of associations (Overall positivity) | 2.67(1.53) | 2.87 (1.60) | –.017 (–.081) | –.013 (–.094) |
| Valence of attitudes about the coronavirus (Overall positivity) | 1.75(0.79) | 1.85 (0.79) | –.028 (–.065) | .063 (.028) |

*Note.* ^+^*p* < 0.1 (2-tailed); *** *p* < 0.05 (2-tailed); ** *p* < 0.01 (2-tailed). Figures in parentheses in the last two columns indicate parameters when controlling for socially desirable responding of participants. Valence of associations was measured on a seven-point Likert scale and Valence of attitudes about the coronavirus was measured on a five-point Likert scale.

**Table 12**

*Valence of associations, valence of attitudes about the coronavirus and green self-identity: Descriptive statistics and correlations*

| Variable |  | |  | |
| --- | --- | --- | --- | --- |
|  | *M (SD)*  *(Wave 1)* | *M (SD)*  *(Wave 2)* | *Correlation with green self-identity*  *(Wave 1)* | *Correlation with green self-identity*  *(Wave 2)* |
| Valence of associations (Overall positivity) | 2.67(1.53) | 2.87 (1.60) | .235** (.146**) | .189** (.131**) |
| Valence of attitudes about the coronavirus (Overall positivity) | 1.75(0.79) | 1.85 (0.79) | .105* (.043) | .209** (.183**) |

*Note.* ^+^*p* < 0.1 (2-tailed); *** *p* < 0.05 (2-tailed); ** *p* < 0.01 (2-tailed). Figures in parentheses in the last two columns indicate parameters when controlling for socially desirable responding of participants. Valence of associations was measured on a seven-point Likert scale and Valence of attitudes about the coronavirus was measured on a five-point Likert scale.

**Table 13**

*Transcendental beliefs about the pandemic and nature connection index: Descriptive statistics and correlations*

| Variable |  | |  | |
| --- | --- | --- | --- | --- |
|  | *M (SD)*  *(Wave 1)* | *M (SD)*  *(Wave 2)* | *Correlation with nature connection index (Wave 1)* | *Correlation with nature connection index (Wave 2)* |
| Coronavirus has come to tell us we are not the kings of the world. | 3.69 (2.13) | 3.77 (2.13) | .184** (.149**) | .141** (.083) |
| Coronavirus is a punishment for mankind that has lost its way. | 3.19 (1.97) | 3.08 (1.98) | .082 (.041) | .039 (–.045) |
| Coronavirus is a way of nature warning us to stop destroying our planet. | 3.41 (2.02) | 3.52 (2.03) | .174** (.143**) | .156** (.094*) |

*Note.* ^+^*p* < 0.1 (2-tailed); *** *p* < 0.05 (2-tailed); ** *p* < 0.01 (2-tailed). Figures in parentheses in the last two columns indicate parameters when controlling for socially desirable responding of participants. All items were measured on a seven-point Likert scale.

**Table 14**

*Transcendental beliefs about the pandemic and green self-identity: Descriptive statistics and correlations*

| Variable |  | |  | |
| --- | --- | --- | --- | --- |
|  | *M (SD)*  *(Wave 1)* | *M (SD)*  *(Wave 2)* | *Correlation with green self-identity*  *(Wave 1)* | *Correlation with green self-identity*  *(Wave 2)* |
| Coronavirus has come to tell us we are not the kings of the world. | 3.69(2.13) | 3.77 (2.13) | .318** (.269**) | .233** (.187**) |
| Coronavirus is a punishment for mankind that has lost its way. | 3.19(1.97) | 3.08 (1.98) | .292** (.238**) | .182** (.118**) |
| Coronavirus is a way of nature warning us to stop destroying our planet. | 3.41(2.02) | 3.52 (2.03) | .384** (.349**) | .312** (.267**) |

*Note.* ^+^*p* < 0.1 (2-tailed); *** *p* < 0.05 (2-tailed); ** *p* < 0.01 (2-tailed). Figures in parentheses in the last two columns indicate parameters when controlling for socially desirable responding of participants. All items were measured on a seven-point Likert scale.

**Table 15**

*The perceived origin of the coronavirus and nature connection index: Descriptive statistics, reliability coefficients of relevant scales and correlations*

| Variable |  | |  | | | |  | |  |
| --- | --- | --- | --- | --- | --- | --- | --- | --- | --- |
|  | *M (SD)*  *(Wave 1)* | *M (SD)*  *(Wave 2)* | | *α*  *(Wave 1)* | *α*  *(Wave 2)* | *Correlation with nature connection index (Wave 1)* | | *Correlation with nature connection index (Wave 2)* | |
| Natural cause | 3.27 (1.77) | 3.41 (1.77) | | .87 | .87 | .006 (–.023) | | –.125** (–.139**) | |
| Human cause | 4.99 (1.69) | 4.80 (1.73) | | .87 | .89 | .089* (.080) | | .197** (.150**) | |

*Note.* ^+^*p* < 0.1 (2-tailed); *** *p* < 0.05 (2-tailed); ** *p* < 0.01 (2-tailed). Figures in parentheses in the last two columns indicate parameters when controlling for socially desirable responding of participants. All items were measured on a seven-point Likert scale.

**Table 16**

*The perceived origin of the coronavirus and green self-identity: Descriptive statistics, reliability coefficients of relevant scales and correlations*

| Variable |  | |  | | | |  | |  |
| --- | --- | --- | --- | --- | --- | --- | --- | --- | --- |
|  | *M (SD)*  *(Wave 1)* | *M (SD)*  *(Wave 2)* | | *α*  *(Wave 1)* | *α*  *(Wave 2)* | *Correlation with green self-identity*  *(Wave 1)* | | *Correlation with green self-identity*  *(Wave 2)* | |
| Natural cause | 3.27 (1.77) | 3.41 (1.77) | | .87 | .87 | .209** (.171**) | | .111* (.108*) | |
| Human cause | 4.99 (1.69) | 4.80 (1.73) | | .87 | .89 | .042 (.023) | | .104* (.057) | |

*Note.* ^+^*p* < 0.1 (2-tailed); *** *p* < 0.05 (2-tailed); ** *p* < 0.01 (2-tailed). Figures in parentheses in the last two columns indicate parameters when controlling for socially desirable responding of participants. All items were measured on a seven-point Likert scale.

Behavioral Tendencies

**Table 17**

*Self-efficacy, compliance with the safety measures, preparedness to act and nature connection index: Descriptive statistics, reliability coefficients of relevant scales and correlations*

| Variable |  | |  | | | |  | |  |
| --- | --- | --- | --- | --- | --- | --- | --- | --- | --- |
|  | *M (SD)*  *(Wave 1)* | *M (SD)*  *(Wave 2)* | | *α*  *(Wave 1)* | *α*  *(Wave 2)* | *Correlation with nature connection index (Wave 1)* | | *Correlation with nature connection index (Wave 2)* | |
| Self-efficacy | 3.65(0.92) | 3.52 (0.98) | | .82 | .86 | .202** (.216**) | | .193** (.168**) | |
| Compliance with the safety measures | 6.41(0.84) | 6.25 (0.97) | | .77 | .78 | .290** (.294**) | | .280** (.278**) | |
| Preparedness to act | 4.19(0.95) | 4.08 (0.96) | | - | - | .173** (.163**) | | .145** (.125**) | |

*Note.* ^+^*p* < 0.1 (2-tailed); *** *p* < 0.05 (2-tailed); ** *p* < 0.01 (2-tailed). Figures in parentheses in the last two columns indicate parameters when controlling for socially desirable responding of participants. Self-efficacy and Preparedness to act were measured on a five-point Likert scale. Compliance with the safety measures was measured on a seven-point Likert scale.

**Table 18**

*Self-efficacy, compliance with the safety measures, preparedness to act and green self-identity: Descriptive statistics, reliability coefficients of relevant scales and correlations*

| Variable |  | |  | | | |  | |  |
| --- | --- | --- | --- | --- | --- | --- | --- | --- | --- |
|  | *M (SD)*  *(Wave 1)* | *M (SD)*  *(Wave 2)* | | *α*  *(Wave 1)* | *α*  *(Wave 2)* | *Correlation with green self-identity*  *(Wave 1)* | | *Correlation with green self-identity*  *(Wave 2)* | |
| Self-efficacy | 3.65(0.92) | 3.52 (0.98) | | .82 | .86 | .037 (.057) | | .150** (.125**) | |
| Compliance with the safety measures | 6.41(0.84) | 6.25 (0.97) | | .77 | .78 | .100* (.103*) | | .201** (.195**) | |
| Preparedness to act | 4.19(0.95) | 4.08 (0.96) | | - | - | .141** (.126**) | | .193** (.176**) | |

*Note.* ^+^*p* < 0.1 (2-tailed); *** *p* < 0.05 (2-tailed); ** *p* < 0.01 (2-tailed).). Figures in parentheses in the last two columns indicate parameters when controlling for socially desirable responding of participants. Self-efficacy and Preparedness to act were measured on a five-point Likert scale. Compliance with the safety measures was measured on a seven-point Likert scale.

**Table 19**

*Activities people engage in while home and nature connection index: Frequencies and logistic regression coefficients*

| Variable | |  | |  | |  | |  |
| --- | --- | --- | --- | --- | --- | --- | --- | --- |
|  | *Frequency (%)*  *(Wave 1)* | | *Frequency (%)*  *(Wave 2)* | | *Β*  *(Wave 1)* | | *Β*  *(Wave 2)* | |
| Watching movies and TV shows | 84.5 | | 79.6 | | .151* (.181*) | | .038 (.005) | |
| Gardening (incl. interior plants) | 15.6 | | 27.3 | | .260** (.243**) | | .315** (.300**) | |
| Reading | 50.7 | | 55.6 | | .193** (.189**) | | .098 (.057) | |
| Social media | 66.1 | | 65.9 | | –.008 (–.014) | | .052 (.018) | |
| Going for a walk / hike in nature | 27.1 | | 32.8 | | .284** (.308**) | | .235** (.277**) | |
| Sport / exercising (indoor) | 26.7 | | 32.2 | | .115^+^ (.080) | | .212** (.117*) | |
| Sport / exercising (outdoor) | 8.8 | | 14.0 | | .173 (.193) | | .264** (.313**) | |
| Eating healthy | 37.4 | | 40.1 | | .219** (.174**) | | .256** (.211**) | |
| Talking to relatives and friends | 53.8 | | 47.9 | | .087 (.087) | | .117^+^ (.134*) | |

*Note.* ^+^*p* < 0.1 (2-tailed); *** *p* < 0.05 (2-tailed); ** *p* < 0.01 (2-tailed). Figures in parentheses in the last two columns indicate parameters when controlling for socially desirable responding of participants.

**Table 20**

*Activities people engage in while home and green self-identity: Frequencies and logistic regression coefficients*

| Variable | |  | |  | |  | |  |
| --- | --- | --- | --- | --- | --- | --- | --- | --- |
|  | *Frequency (%)*  *(Wave 1)* | | *Frequency (%)*  *(Wave 2)* | | *Β*  *(Wave 1)* | | *Β*  *(Wave 2)* | |
| Watching movies and TV shows | 84.5 | | 79.6 | | .188^+^ (.261*) | | .221* (.199*) | |
| Gardening (incl. interior plants) | 15.6 | | 27.3 | | .371** (.363**) | | .242* (.213*) | |
| Reading | 50.7 | | 55.6 | | .294** (.310**) | | .273** (236**) | |
| Social media | 66.1 | | 65.9 | | .116 (.119) | | .225** (198*) | |
| Going for a walk / hike in nature | 27.1 | | 32.8 | | .137 (.196*) | | –.009 (.012) | |
| Sport / exercising (indoor) | 26.7 | | 32.2 | | .240** (.174^+^) | | .442** (.409**) | |
| Sport / exercising (outdoor) | 8.8 | | 14.0 | | .149 (.142) | | .027 (.061) | |
| Eating healthy | 37.4 | | 40.1 | | .486** (.407**) | | .356** (.302**) | |
| Talking to relatives and friends | 53.8 | | 47.9 | | –.020 (–.035) | | .030 (.040) | |

*Note.* ^+^*p* < 0.1 (2-tailed); *** *p* < 0.05 (2-tailed); ** *p* < 0.01 (2-tailed). Figures in parentheses in the last two columns indicate parameters when controlling for socially desirable responding of participants.

Perceived Impact of the Pandemic

**Table 21**

*Perceived threat and nature connection index: Descriptive statistics and correlations*

| Variable |  | |  | |
| --- | --- | --- | --- | --- |
|  | *M (SD)*  *(Wave 1)* | *M (SD)*  *(Wave 2)* | *Correlation with nature connection index (Wave 1)* | *Correlation with nature connection index (Wave 2)* |
| The coronavirus is a threat to you personally. | 4.78(1.84) | 4.66 (1.84) | .276** (.264**) | .117** (.087*) |
| The coronavirus is a threat to people around you. | 5.34(1.61) | 5.16 (1.66) | .233** (.231**) | .141** (.135**) |
| The coronavirus is a threat to people in your country | 5.76(1.39) | 5.53 (1.44) | .264** (.280**) | .217** (.206**) |
| The coronavirus is a threat to humans in general. | 5.79(1.42) | 5.64 (1.46) | .239** (.253**) | .211** (.198**) |
| The coronavirus is a threat to the natural environment in general. | 3.45(2.02) | 3.57 (2.09) | .095* (.036) | .118** (.030) |
| The coronavirus is a threat to the natural environment in your country. | 3.56(2.11) | 3.61 (2.10) | .126** (.070) | .119** (.032) |
| The coronavirus is a threat to the natural environment around you. | 3.45(2.09) | 3.58 (2.12) | .092* (.028) | .100* (.011) |

*Note.* ^+^*p* < 0.1 (2-tailed); *** *p* < 0.05 (2-tailed); ** *p* < 0.01 (2-tailed). Figures in parentheses in the last two columns indicate parameters when controlling for socially desirable responding of participants. All items were measured on a seven-point Likert scale.

**Table 22**

*Perceived threat and green self-identity: Descriptive statistics and correlations*

| Variable |  | |  | |
| --- | --- | --- | --- | --- |
|  | *M (SD)*  *(Wave 1)* | *M (SD)*  *(Wave 2)* | *Correlation with green self-identity*  *(Wave 1)* | *Correlation with green self-identity*  *(Wave 2)* |
| The coronavirus is a threat to you personally. | 4.78 (1.84) | 4.66 (1.84) | .326** (.316**) | .259** (.237**) |
| The coronavirus is a threat to people around you. | 5.34 (1.61) | 5.16 (1.66) | .276** (.283**) | .276** (.275**) |
| The coronavirus is a threat to people in your country | 5.76 (1.39) | 5.53 (1.44) | .243** (.280**) | .291** (.283**) |
| The coronavirus is a threat to humans in general. | 5.79 (1.42) | 5.64 (1.46) | .233** (.269**) | .305** (.296**) |
| The coronavirus is a threat to the natural environment in general. | 3.45 (2.02) | 3.57 (2.09) | .364** (.288**) | .269** (.204**) |
| The coronavirus is a threat to the natural environment in your country. | 3.56 (2.11) | 3.61 (2.10) | .352** (.275**) | .268** (.204**) |
| The coronavirus is a threat to the natural environment around you. | 3.45 (2.09) | 3.58 (2.12) | .353** (.266**) | .269** (.204**) |

*Note.* ^+^*p* < 0.1 (2-tailed); *** *p* < 0.05 (2-tailed); ** *p* < 0.01 (2-tailed). Figures in parentheses in the last two columns indicate parameters when controlling for socially desirable responding of participants. All items were measured on a seven-point Likert scale.

**Table 23**

*Psychological distance and nature connection index: Descriptive statistics and correlations*

| Variable |  | |  | |
| --- | --- | --- | --- | --- |
|  | *M (SD)*  *(Wave 1)* | *M (SD)*  *(Wave 2)* | *Correlation with nature connection index (Wave 1)* | *Correlation with nature connection index (Wave 2)* |
| My local area where I live is likely to be affected by the coronavirus pandemic. | 4.03 (1.04) | 3.91 (1.06) | .143** (.167**) | .115** (.164**) |
| The coronavirus pandemic will mostly affect areas that are far away from where I live. | 2.94 (1.50) | 3.02 (1.44) | .038 (.012) | .024 (–.008) |
| The coronavirus pandemic is likely to have a big impact on people like me. | 3.68 (1.12) | 3.59 (1.13) | .222** (.225**) | .131** (.125**) |
| People very different from me are going to be greatly affected by the coronavirus pandemic. | 3.85 (1.09) | 3.85 (1.04) | .080 (.085) | .097* (.126*) |
| Please indicate how familiar you are with the closest person in your environment that is directly affected by the coronavirus. | 2.56 (1.44) | 2.81 (1.46) | .013 (–.008) | .023 (.009) |
| When will the coronavirus pandemic start affecting your personal life? | 5.84 (1.62) | 5.69 (1.68) | .190** (.207**) | .137** (.185**) |
| When will the coronavirus pandemic start affecting the lives of people in your area? | 6.11 (1.35) | 6.03 (1.41) | .184** (.213**) | .197** (.247**) |
| What do you think is the likelihood (in %) that you personally will contract the coronavirus | 41.45 (29.14) | 38.56 (28.96) | .107* (.093*) | –.024 (–.036) |
| What do you think is the likelihood (in %) that people in your area will contract the coronavirus? | 59.72 (31.08) | 55.96 (30.24) | .089* (.099*) | .035 (.048) |

*Note.* ^+^*p* < 0.1 (2-tailed); *** *p* < 0.05 (2-tailed); ** *p* < 0.01 (2-tailed). Figures in parentheses in the last two columns indicate parameters when controlling for socially desirable responding of participants. Geographical and social distance were measured on a five-point Likert scale. Temporal distance was measured on a seven-point Likert scale. Hypothetical distance was measured as percentage out of 100.

**Table 24**

*Psychological distance and green self-identity: Descriptive statistics and correlations*

| Variable |  | |  | |
| --- | --- | --- | --- | --- |
|  | *M (SD)*  *(Wave 1)* | *M (SD)*  *(Wave 2)* | *Correlation with green self-identity*  *(Wave 1)* | *Correlation with green self-identity*  *(Wave 2)* |
| My local area where I live is likely to be affected by the coronavirus pandemic. | 4.03 (1.04) | 3.91 (1.06) | .041 (.082) | .161** (.205**) |
| The coronavirus pandemic will mostly affect areas that are far away from where I live. | 2.94 (1.50) | 3.02 (1.44) | .116* (.073) | .043 (.016) |
| The coronavirus pandemic is likely to have a big impact on people like me. | 3.68 (1.12) | 3.59 (1.13) | .234** (.249**) | .161** (.156**) |
| People very different from me are going to be greatly affected by the coronavirus pandemic. | 3.85 (1.09) | 3.85 (1.04) | .016 (.023) | .060 (.085) |
| Please indicate how familiar you are with the closest person in your environment that is directly affected by the coronavirus. | 2.56 (1.44) | 2.81 (1.46) | .227** (.206**) | .175** (.168**) |
| When will the coronavirus pandemic start affecting your personal life? | 5.84 (1.62) | 5.69 (1.68) | –.004 (.019) | .070 (.109*) |
| When will the coronavirus pandemic start affecting the lives of people in your area? | 6.11 (1.35) | 6.03 (1.41) | –.038 (.004) | .095* (.135**) |
| What do you think is the likelihood (in %) that you personally will contract the coronavirus | 41.45 (29.14) | 38.56 (28.96) | .197** (.183**) | .178** (.175**) |
| What do you think is the likelihood (in %) that people in your area will contract the coronavirus? | 59.72 (31.08) | 55.96 (30.24) | .056 (.074) | .149** (.164**) |

*Note.* ^+^*p* < 0.1 (2-tailed); *** *p* < 0.05 (2-tailed); ** *p* < 0.01 (2-tailed). Figures in parentheses in the last two columns indicate parameters when controlling for socially desirable responding of participants. Geographical and social distance were measured on a five-point Likert scale. Temporal distance was measured on a seven-point Likert scale. Hypothetical distance was measured as percentage.

Word Association Task

| Category | Description | Wave 1  Mentioned (1 = Yes ; 0 = No) | Wave 2  Mentioned (1 = Yes ; 0 = No) |
| --- | --- | --- | --- |
| Situation related | e.g. virus, pandemic, infection, disease, Wuhan, sickness, worldwide | 420 | 420 |
| Symptoms | e.g. fever, cough, pain | 109 | 80 |
| General health | e.g. doctors, hospitals, medicine, flu, pneumonia, health | 151 | 163 |
| Measures | e.g. masks, hand washing, quarantine, social distance | 203 | 255 |
| Economy | e.g. market money, economic crises, job loss | 72 | 109 |
| Social | e.g. family, elderly, human, people | 61 | 57 |
| Emotions (uncertainty) | e.g. confusion, risk, strange, uncertainty | 33 | 61 |
| Emotions (anger) | e.g. hate, upset, pissed | 21 | 17 |
| Emotions (anxiety) | e.g. stress, panic, overaction | 89 | 55 |
| Emotions (fear) | e.g. scared, fear, concerned, worried | 158 | 140 |
| Emotions (sad) | e.g. depressing, sad | 47 | 64 |
| Emotions (loneliness) | e.g. isolation, loneliness, alone | 80 | 82 |
| Politics | e.g. Trump, government | 41 | 39 |
| Nature related | e.g. less pollution, good for the environment | 5 | 8 |
| Death related | e.g. death, dying | 227 | 223 |

Good things about the coronavirus

| Category | Description | Wave 1  Mentioned (1 = Yes ; 0 = No) | Wave 2  Mentioned (1 = Yes ; 0 = No) |
| --- | --- | --- | --- |
| Social contact | e.g. family, more time with family, kids | 84 | 123 |
| Global community | e.g. helping each other | 87 | 75 |
| Reflection | e.g. grateful, appreciative, reevaluate life, spiritual, stop, normal life | 47 | 64 |
| Health | e.g. cooking food, doing sport, hygienic, taking care of myself | 41 | 38 |
| Work | e.g. working from home | 8 | 13 |
| Nature related | e.g. less pollution, good for the environment | 114 | 154 |
| Death related | e.g. death, dying | 12 | 6 |

Bad things about the coronavirus

| Category | Description | Wave 1  Mentioned (1 = Yes ; 0 = No) | Wave 2  Mentioned (1 = Yes ; 0 = No) |
| --- | --- | --- | --- |
| Situation related | e.g. virus, pandemic, infection, disease, Wuhan, sickness, worldwide | 394 | 213 |
| Symptoms | e.g. fever, cough, pain | 13 | 11 |
| General health | e.g. doctors, hospitals, medicine, flu, pneumonia, health | 68 | 50 |
| Measures | e.g. masks, hand washing, quarantine, social distance | 68 | 83 |
| Economy | e.g. market money, economic crises, job loss | 251 | 271 |
| Social | e.g. family, elderly, human, people | 192 | 194 |
| Emotions (uncertainty) | e.g. confusion, risk, strange, uncertainty | 19 | 19 |
| Emotions (anger) | e.g. hate, upset, pissed | 4 | 6 |
| Emotions (anxiety) | e.g. stress, panic, overaction | 37 | 29 |
| Emotions (fear) | e.g. scared, fear, concerned, worried | 29 | 37 |
| Emotions (sad) | e.g. depressing, sad | 12 | 23 |
| Emotions (loneliness) | e.g. isolation, loneliness, alone | 39 | 43 |
| Politics | e.g. Trump, government | 24 | 30 |
| Nature related | e.g. less pollution, good for the environment | 2 | 6 |
| Death related | e.g. death, dying | 334 | 340 |

Description of all the scales used in the study (waves 1 & 2)

| Measure | Source | Items | Response format | Cronbach’s alpha | Results (Themes) |
| --- | --- | --- | --- | --- | --- |
| Extended Inclusion of Nature in Self scale | Martin & Czellar (2016) | 1.“Please choose the picture below that best describes your relationship with the natural environment.”  2.“Please choose the picture below that best describes nature when you think of your relationship with the natural environment.”  3.“Please choose the picture below that best describes your relationship with the natural environment.”  4.“Please choose the picture below that best describes your relationship with the natural environment.” | 1 (distant) – 7 (close) graphical response options illustrating the relationship of the self with nature. | α = .84 (Wave 1)  α = .88 (Wave 2) | All |
| Nature connection index Scale | Richardson et al. (2019) | 1.“I always find beauty in nature.”  2. “I always treat nature with respect.”  3. “Being in nature makes me very happy.”  4. “Spending time in nature is very important to me.”  5. “I find being in nature really amazing.”  6. “I feel part of nature.” | 1 (completely disagree) – 9 (completely agree) response options | α = .93 (Wave 1)  α = .92 (Wave 2) | All |
| Green self-identity | Sparks, & Shepherd (1992) | 1. “I think of myself as a "green consumer."  2. “I think of myself as someone who is very concerned with "green issues."” | 1 (completely disagree) – 5 (completely agree) response options | α = .84 (Wave 1)  α = .86 (Wave 2) | All |
| Salience of self-nature relationship in daily life | Self-constructed (based on categories from Schultz & Kaiser, 2012) | “In the following aspects of your daily life, how often do you think about your relationship with the natural environment?”  1. “House-related activities”  2. “Activities related to transportation and traveling”  3. “Activities related to waste disposal”  4. “Consumption-related activities” | 1 (never) – 7 (very often) response options | α = .82 (Wave 1)  α = .85 (Wave 2) | - |
| Power of Nature scale | Self-constructed | 1. “I feel that the people in my country have power over nature.”  2. “I feel that I have power over nature.”  3. “I feel that nature has power over the people in my country.”  4. “I feel that nature has power over me.” | 1 (not at all) – 9 (very much) response options | α = .66 (Wave 1)  α = .70 (Wave 2) | - |
| Frequency of pro-environmental behaviors | Tam (2013)  + 2 additional high impact pro-environmental behaviors (13. & 14.) | “Please evaluate how frequently you perform the following behaviors in daily life.”  1. “Looking for ways to reuse things.”  2. “Recycling things (e.g., papers, cans or bottles).”  3. “Encouraging friends or family to recycle.”  4. “Purchasing products in reusable containers.”  5. “Writing a letter to public authorities to support an environmental issue.”  6. “Volunteering time to help an environmentalist group.”  7. “Buying environmentally friendly products even if they may not work as well as competing products.”  8. “Purchasing something made of recycled materials even though it is more expensive.”  9. “Buying products only from companies that have a strong record of protecting the environment.”  10. “Contacting public authorities to complain about environmental problems.”  11. “Taking a shorter shower to conserve water.”  12. “Using energy-efficient household devices such as light bulbs.”  13. “Living car-free.”  14. “Eating less meat.” | 1 (never) – 7 (very often) response options | α = .90 (Wave 1)  α = .89 (Wave 2) | - |
| Pro-environmental donations | Self-constructed | “On average, how much do you donate to support pro-environmental organizations?” | Slider: 1 – 1000 $ response option with 1$ unit |  | - |
| Other pro-environmental behaviors | Self-constructed | “What other sustainable behaviors do you engage in?” | Text box response options (10) |  | - |
| Association task | Adapted from: Lorenzoni, Leiserowitz, de Franca Doria, Poortinga, & Pidgeon, 2006 | “Please write down all the words that come to your mind when you think about 'CORONAVIRUS'. Use one box per word and use up as many boxes as you can/like.”  “Please evaluate the words that came to your mind when you thought about 'CORONAVIRUS' on a scale from highly negative to highly positive.” | Text box response options (10)  1 (highly negative) – 4 (neutral) – 7 (highly positive) response options |  | Individual Representations |
| Perception of threat | Adapted from: Bord, Fisher, & Robert (1998), and extended | “Please indicate how much you perceive the coronavirus pandemic to be a threat ...”  1. “... to you personally”  2. “... to people around you”  3. “... to people in your country”  4. “... to humans in general”  1. “... to the natural environment in general”  2. “... to the natural environment in your country”  3. “... to the natural environment around you” | 1 (not at all) – 7 (very much) response options |  | Perceived Impact of the Pandemic |
| Perception of good and bad things | Adapted from: Spence, Poortinga, Pidgeon, & Lorenzoni (2010), and shortened | 1. “There are a lot of bad things about the coronavirus pandemic.”  “Please give us some examples of the bad things.”  2. “There are a lot of good things about the coronavirus pandemic.”  “Please give us some examples of the good things.” | 1 (strongly disagree) – 5 (strongly agree) response options  +  Text box response option |  | Individual Representations |
| Beliefs about possible causes | Adapted from: Heath & Gifford, 2006 | 1. “The coronavirus pandemic is mainly due to natural causes, not human activity.”  2. “The main causes of the coronavirus pandemic are human activities.”  3. “Coronavirus pandemic is merely a natural fluctuation, not caused by human activity.”  4. “I am quite sure that human activities are to be blamed for the coronavirus pandemic.” | 1 (strongly disagree) – 7 (strongly agree) response options | Nature-cause index: α = .87 (Wave 1)  Human-cause index: α = .87 (Wave 1)  Nature-cause index: α = .87 (Wave 2)  Human-cause index: α = .89 (Wave 2) | Individual Representations |
| Self-efficacy | Adapted from: Heath & Gifford, 2006 | 1. “There are simple things that I can do that will have a meaningful effect to alleviate the negative effects of the coronavirus pandemic.”  2. “I believe that the little things I can do will make a difference to alleviate the negative effects of the coronavirus pandemic.”  3.“Even if I try to do something about the coronavirus pandemic, I doubt it will make any difference.”  4. “There is very little I can do to mitigate the negative effect of the coronavirus pandemic.” | 1 (strongly disagree) – 5 (strongly agree) response options | α = .82 (Wave 1)  α = .86 (Wave 1) | Behavioral Tendencies |
| Preparedness to act | Adapted from: Heath & Gifford, 2006 | “I am taking concrete steps to do something to mitigate the negative effects of the coronavirus pandemic.”  “Please give us some concrete examples of the steps you are taking.” | 1 (strongly disagree) – 5 (strongly agree) response options  +  Text box response option |  | Behavioral Tendencies |
| Geographical distance | Adapted from: Spence, Poortinga, & Pidgeon (2012) | 1. “My local area where I live is likely to be affected by the coronavirus pandemic.”  2. “The coronavirus pandemic will mostly affect areas that are far away from where I live.” | 1 (strongly disagree) – 5 (strongly agree) response options |  | Perceived Impact of the Pandemic |
| Social distance | Adapted from: Spence, Poortinga, & Pidgeon (2012) and extended, adjusted | 1. “The coronavirus pandemic is likely to have a big impact on people like me.”  2. “People very different from me are going to be greatly affected by the coronavirus pandemic.” | 1 (strongly disagree) – 5 (strongly agree) response options |  | Perceived Impact of the Pandemic |
| Familiarity with closest affected | Self-constructed | “Please indicate how familiar you are with the closest person in your environment that is directly affected by the coronavirus.” | 1 (not at all familiar) – 5 (very familiar) response options |  | Perceived Impact of the Pandemic |
| Temporal distance | Adapted from Spence, Poortinga, & Pidgeon (2012) and extended, adjusted | 1. “When will the coronavirus pandemic start affecting your personal life?”  2. “When will the coronavirus pandemic start affecting the lives of people in your area?” | 1 (never) – 7 (it is already affecting it) response options |  | Perceived Impact of the Pandemic |
| Hypothetical distance | Self-constructed | 1. “What do you think is the likelihood (in %) that you personally will contract the coronavirus?”  2. “What do you think is the likelihood (in %) that people in your area will contract the coronavirus?” | Slider: 1 – 100 % response option with 1% unit |  | Perceived Impact of the Pandemic |
| Present and future orientation | Self-constructed | 1. “I am focused on the ... “  2. “I feel ...”  3. “I feel ...” | 1 (immediate present) – 9 (distant future)  1 (not at all present-oriented– 9 (very much present-oriented)  1 (not at all future-oriented) – 9 (very much future-oriented) response options |  | - |
| Activities |  | “Please tick off those activities that you engage in more during the coronavirus pandemic than you usually do. Please check all that apply:” | Multiple choice + text box response options:  - Watching movies and TV shows  - Gardening (incl. interior plants)  - Reading  - Social media  - Going for a walk/hike in nature  - Sports/ exercising - indoors  - Sports/ exercising outdoors  - Eating healthy  - Talking to relatives and friends  - Other / Additional activities |  | Behavioral Tendencies |
| Compliance with health safety measures | Self-constructed | “How likely are you to engage in the following behaviors?”  1. “Washing your hands more frequently and thoroughly.”  2. “Staying at home rather than going out in public, if not absolutely necessary.”  3. “Keeping distance from other people (social distancing) of at least 6 feet/2 meters.”  4. “Staying informed and following the news.” | 1 (not at all likely) – 7 (very likely) response options | α = .77 (Wave 1)  α = .78 (Wave 2) | Behavioral Tendencies |
| Practicing self-quarantine | Self-constructed | “As a response to the pandemic, do you currently practice self-quarantine for you and your family?  It means completely staying at home except for shopping and the occasional walk, and complete restriction of contacts to people outside the household.”  If yes  “For how many days now?” | Yes / No response options  Text box |  | Behavioral Tendencies |
| Shopping choices | Self-constructed | “Imagine you are shopping at the supermarket in this very moment and have to decide between various products. Which products would you choose to buy?” | Binary response options (pictures):  Organic vs. Conventional apple  Cleaned vs. unclean carrots  Cucumber packed in plastic vs. not packed  Bio vs. non-bio detergent |  | - |
| Time spent on social media per day | Self-constructed | “How many hours per day do you spend on social networks?” | Slider: 1 – 24 hours response option with 1 hour unit |  | - |
| Beliefs about the meaning of the pandemic | Self-constructed (inspired by popular media and blog posts) | “Please indicate how much do you agree with the following statements:”  1. “Coronavirus has come to tell us we are not the kings of the world.”  2. “Coronavirus is a punishment for mankind that has lost its way.”  3. “Coronavirus is a way of nature warning us to stop destroying our planet.” | 1 (strongly disagree) – 7 (strongly agree) response options |  | Individual Representations |
|  |  |  |  |  |  |
| Social desirability | Adapted from Podsakoff, et al. (2003) | 1. “I never regret my decisions.”  2. “I am very confident in my judgements.”  3. “When I hear people talking privately, I avoid listening.”  4. “I don't gossip about other people's business.” | 1 (strongly disagree) – 7 (strongly agree) response options |  | All |

Description of the additional scales used in the study (only wave 2)

| Measure | Source | Items | Response format | Cronbach’s alpha |
| --- | --- | --- | --- | --- |
| Climate change concern | (Spence et al., 2012) | 1.“How concerned are you, if at all, about climate change, sometimes referred to as "global warming"?”  2.“Considering any potential effects of climate change which there might be on you personally, how concerned, if at all, are you about climate change?”  3.“Considering any potential effects of climate change there might be on society in general, how concerned are you about climate change?” | 1 (not at all concerned) – 4 (very concerned) response options | α = .95 |
| Belief in environment improvements | Self-constructed | “Please indicate to what extent you agree with the following statements:”  1.“The current pandemic situation will have a lasting positive effect on the environment because of the measures taken to contain the virus.”  2. “The current pandemic situation will have only a temporary positive effect on the environment because of the measures taken to contain the virus.” | 1 (strongly disagree) – 7 (strongly agree) response options |  |
| Plans after the coronavirus pandemic | Self-constructed | “Please fill the "BLANKS" in the following statements with the response option that best applies.  After the coronavirus pandemic is over, I want to ...”  1. “travel (*blank)* in comparison to the times before the pandemic”  2. “take *(blank)* long-distance trips in comparison to the times before the pandemic”  3. “use *(blank)* public transport in comparison to the times before the pandemic”  4. “drive my car *(blank)* in comparison to the times before the pandemic”  5. “consume *(blank)* sustainably in comparison to the times before the pandemic”  6. “produce *(blank)* food waste in comparison to the times before the pandemic”  7. “shop online *(blank)* in comparison to the times before the pandemic” | Blanks: “less”, “about the same”, “more” |  |
| Spiritual oneness | (Garfield et al., 2014) | “Please indicate to what extent do you agree with the following statements:”.  1. “There is a unifying force (in the universe) through which all life is brought together in one great whole.”  2. “There is a mysterious link, beyond the purely physical, that connects all human beings with each other and with the entire natural world.”  3. “A vital thread of life joins all objects and beings in the universe.”  4. “Human beings and nature are both part of a vast symphony of life directed by a single life-force.”  5. “The peace and happiness of humankind is founded on being in harmony with the rhythm of the universe.”  6. “All existence in the universe forms one great unified life system.”  7. “The natural world does not consist merely of physical phenomena but contains spiritual and emotional elements as well.”  8. “Every living and nonliving thing is an expression of the fundamental life-force of the entire cosmos.” | 1 (strongly disagree) – 9 (strongly agree) response options | α = .96 |

References

Bord, R. J., Fisher, A., & Robert, E. O. (1998). Public perceptions of global warming: United States and international perspectives. *Climate Research*, 11(1), 75-8.

Garfield, A. M., Drwecki, B. B., Moore, C. F., Kortenkamp, K. V., & Gracz, M. D. (2014). The oneness beliefs scale: Connecting spirituality with pro-environmental behavior. *Journal for the Scientific Study of Religion*, 53(2), 356–372.

Heath, Y., & Gifford, R. (2006). Free-market ideology and environmental degradation: The case of belief in global climate change*. Environment and Behavior*, 38(1), 48-71.

Martin, C., & Czellar, S. (2016). The extended Inclusion of Nature in Self scale. *Journal of Environmental Psychology*, 47, 181–194.

Lorenzoni, I., Leiserowitz, A., de Franca Doria, M., Poortinga, W., & Pidgeon, N. F. (2006). Cross‐National comparisons of image associations with “global warming” and “climate change” among laypeople in the United States of America and Great Britain. *Journal of Risk Research*, 9(03), 265-281.

Podsakoff, P. M., MacKenzie, S. B., Lee, J. Y., & Podsakoff, N. P. (2003). Common method biases in behavioral research: A critical review of the literature and recommended remedies*. Journal of Applied Psychology*, 88, 879-903.

Richardson, M., Hunt, A., Hinds, J., Bragg, R., Fido, D., Petronzi, D., ... & White, M. (2019). A measure of nature connectedness for children and adults: Validation, performance, and insights. *Sustainability*, 11(12), 3250.

Sparks, P., & Shepherd, R. (1992). Self-identity and the theory of planned behavior: Assessing the role of identification with “green consumerism”. *Social Psychology Quarterly*, 55, 388 - 399.

Spence, A., Poortinga, W., & Pidgeon, N. (2012). The psychological distance of climate change. *Risk Analysis: An International Journal*, *32*(6), 957-972

Spence, A., Poortinga, W., Pidgeon, N., & Lorenzoni, I. (2010). Public perceptions of energy choices: The influence of beliefs about climate change and the environment. *Energy & Environment*, *21*(5), 385-407.
